# Manipulating the EphB4-ephrinB2 axis to reduce metastasis in HNSCC

**DOI:** 10.1038/s41388-024-03208-9

**Published:** 2024-11-03

**Authors:** Khalid N. M. Abdelazeem, Diemmy Nguyen, Sophia Corbo, Laurel B. Darragh, Mike W. Matsumoto, Benjamin Van Court, Brooke Neupert, Justin Yu, Nicholas A. Olimpo, Douglas Grant Osborne, Jacob Gadwa, Richard B. Ross, Alexander Nguyen, Shilpa Bhatia, Mohit Kapoor, Rachel S. Friedman, Jordan Jacobelli, Anthony J. Saviola, Michael W. Knitz, Elena B. Pasquale, Sana D. Karam

**Affiliations:** 1 Department of Radiation Oncology, University of Colorado Denver, Anschutz Medical Campus, Aurora, CO USA; 2 Radiation Biology Research Department, National Center for Radiation Research and Technology, Egyptian Atomic Energy Authority, Cairo, Egypt; 3 Department of Immunology and Microbiology, University of Colorado Anschutz Medical Campus, Aurora, CO USA; 4 Cancer Center, Sanford Burnham Prebys Medical Discovery Institute, La Jolla, CA USA; 5 Department of Otolaryngology - Head and Neck Surgery, University of Colorado Anschutz Medical Campus, Aurora, CO USA; 6 Department of Dermatology, University of Colorado Anschutz Medical Campus, Aurora, CO USA; 7 Krembil Research Institute, University Health Network, and University of Toronto, Toronto, ON Canada; 8 Barbara Davis Research Center, University of Colorado Anschutz Medical Campus, Aurora, CO USA; 9 Department of Biochemistry and Molecular Genetics, University of Colorado Anschutz Medical Center, Aurora, CO USA; 10 Department of Radiation Oncology, Washington University School of Medicine, St. Louis, MO USA

**Keywords:** Head and neck cancer, Metastasis

## Abstract

The EphB4-ephrinB2 signaling axis has been heavily implicated in metastasis across numerous cancer types. Our emerging understanding of the dichotomous roles that EphB4 and ephrinB2 play in head and neck squamous cell carcinoma (HNSCC) poses a significant challenge to rational drug design. We find that EphB4 knockdown in cancer cells enhances metastasis in preclinical HNSCC models by augmenting immunosuppressive cells like T regulatory cells (Tregs) within the tumor microenvironment. EphB4 inhibition in cancer cells also amplifies their ability to metastasize through increased expression of genes associated with hallmark pathways of metastasis along with classical and non-classical epithelial-mesenchymal transition. In contrast, vascular ephrinB2 knockout coupled with radiation therapy (RT) enhances anti-tumor immunity, reduces Treg accumulation into the tumor, and decreases metastasis. Notably, targeting the EphB4-ephrinB2 signaling axis with the engineered ligands ephrinB2-Fc-His and Fc-TNYL-RAW-GS reduces local tumor growth and distant metastasis in a preclinical model of HNSCC. Our data suggests that targeted inhibition of vascular ephrinB2 while avoiding inhibition of EphB4 in cancer cells could be a promising strategy to mitigate HNSCC metastasis.

## Introduction

Head and neck squamous cell carcinoma (HNSCC) is a formidable challenge in oncology and stands as the sixth most prevalent cancer type globally with a 5-year survival rate below 50% [1–3]. Despite advances in treatment modalities, the prognosis remains notably poor, particularly for patients with advanced disease. Approximately 10% of HNSCC patients present with distant metastases, and up to 30% additional patients develop distant metastases as their disease progresses [2]. Patients with distant metastases have a median overall survival of only 10 months. These grim statistics underscore the urgent need for innovative therapeutic strategies specifically targeting distant metastases in the context of HNSCC [2].

Metastasis is a complex process through which cancer cells escape from the primary tumor and seed distant sites [4]. The process of metastasis involves a series of sequential steps, including invasion, intravasation into blood or lymphatic vessels, circulation, extravasation at distant sites, and colonization, each of which is influenced by complex interactions between tumor cells, the vascular network, and the immune microenvironment [5]. The tumor vasculature is a physical barrier that can regulate infiltration of cancer and immune cells into the tumor, making it an attractive target to inhibit metastasis [6]. Recent evidence indicates that adhesion and co-stimulatory molecules expressed by the tumor endothelium can promote cancer cell and lymphocyte adhesion, transmigration, or survival, thereby modulating the local and systemic anti-tumor response [7, 8].

Numerous genes have been implicated in metastasis, including the Eph receptor and ephrin gene families [9]. Eph receptors form the largest family of receptor tyrosine kinases (RTKs); they bind to membrane-bound ephrin ligands, and both are capable of signal transduction [10]. Eph receptors and ephrins play crucial roles during embryogenesis, particularly in processes such as axon guidance, cell migration, angiogenesis, and hematopoiesis [9]. In adulthood, Eph receptors and ephrins are expressed during tissue repair, wound healing, and pathologies such as immune diseases and cancer, where they function to mediate neo-angiogenesis, fibrosis, inflammation, and immunosuppression [11–13].

EphB4 and ephrinB2 are a receptor-ligand pair that has been heavily studied for their role in metastasis across numerous cancer types [14–17]. We have reported that within the HNSCC tumor microenvironment (TME), EphB4 is predominantly expressed in cancer cells and its knockdown or knockout drives local progression by increasing immunosuppressive immune cells in the TME [18]. In contrast, ephrinB2 is highly expressed on the vascular endothelium, and its knockout in this compartment suppresses tumor growth, decreases intratumoral infiltration of regulatory T cells (Tregs), and increases activation of CD8 + T cells [18]. These data suggest that cancer cell EphB4 and vascular ephrinB2 play dichotomous roles in HNSCC progression. However, our previous studies were limited to examining the role of EphB4-ephrinB2 signaling in mediating local tumor growth [18, 19]. EphB4 and ephrinB2 have been associated with regional metastasis to the lymph nodes in HNSCC, and distant metastasis often occurs in conjunction with nodal spread [4, 20–23]. Given the prevalence of distant metastases in HNSCC, its significant contribution to patient mortality, and the limited translational therapeutics in that space, we sought to investigate how manipulation of the EphB4-ephrinB2 signaling axis impacts metastatic spread to distant sites.

Ongoing research efforts aim at investigating the impact of manipulating EphB4 and ephrinB2 signaling pathways in metastasis and tumor progression. Our findings reveal that EphB4 knockdown in cancer cells leads to an increase in the development of distant metastases, characterized by a pro-metastatic cancer cell phenotype, systemic immunosuppression, and enhanced infiltration of CD4 + T cells and Tregs into the tumor. We also show that ephrinB2 knockout in the vascular endothelium decreases local tumor growth and metastasis. This effect is mediated by improved effector T cell activity and an increase in T cell expansion in the tumor draining lymph nodes. Finally, we test two novel agents, ephrinB2-Fc-His and Fc-TNYL-RAW-GS, for their ability to overcome distant metastases. Overall, our study underscores the significance of EphB4 and ephrinB2 signaling pathways in HNSCC metastasis and highlights the therapeutic potential of targeting this axis to disrupt metastasis in HPV-unrelated HNSCC.

## Results

### Knockdown of EphB4 in HNSCC cancer cells promotes distant metastasis

**Fig. 1:**
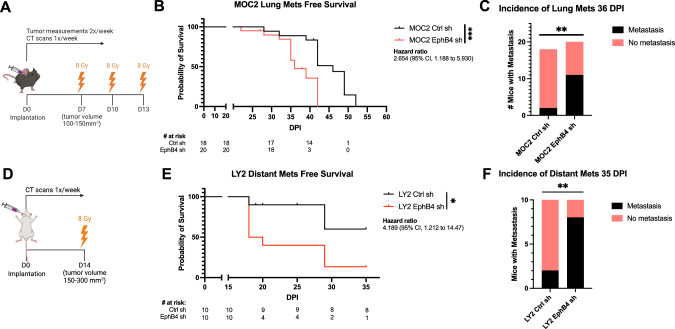
Knockdown of EphB4 in HNSCC cancer cells promotes distant metastasis. **A** Schematic showing experimental design for C57BL/6 J mice implanted with 100k MOC2 cancer cells. 3 fractions of 8 Gray (Gy) radiation therapy (RT) were given as indicated. **B** Kaplan-Meier curves showing lung metastasis free survival of MOC2 control (Ctrl) shRNA (sh) (*n* = 18) versus EphB4 shRNA(*n* = 20) tumors implanted in C57BL/6 J mice. Numbers at risk indicate mice that were alive without metastases at specified timepoints. **C** Contingency table quantifying the incidence of lung metastasis detected by computed tomography (CT) scans in C57BL/6 mice implanted with MOC2 cancer cells by 36 days post-implantation (DPI). **D** Schematic showing experimental design for BALB/c mice implanted with 100k LY2 cancer cells. One fraction of 8 Gy RT was given as indicated. **E** Kaplan-Meier curves showing distant metastasis free survival of LY2 Ctrl shRNA (*n* = 10) vs LY2 EphB4 (*n* = 10) shRNA tumors implanted in BALB/c mice. Numbers at risk indicate mice that were alive without metastases at specified timepoints. **F** Contingency table quantifying the incidence of distant metastases detected by CT scans in BALB/c mice implanted with LY2 Ctrl shRNA or EphB4 shRNA tumors by 35 DPI. The experiments were replicated twice. For Kaplan-Meier survival curves, significance was determined by a log-rank Mantel-Cox test. For contingency tables indicating the incidence of metastases, significance was determined by a Chi-square test. Significance was determined if the *p*-value was < 0.05*, < 0.01**, and < 0.001***. *p*-values are indicated for the figures (**B**) ***p = 0.0009, (**C**) **p = 0.0044, (**E**) *p = 0.0200, (**F**) **p = 0.0073. The error bars represent the standard error of the mean ( ± SEM).

We had previously demonstrated that EphB4 is predominantly expressed in cancer cells in various HPV-negative models of HNSCC and that its knockdown or knockout accelerates local tumor growth in the absence of radiation therapy (RT) [18]. Here, we sought to determine the effects of EphB4 on distant metastasis using two orthotopic models of HNSCC in the context of RT: the MOC2 (C57BL/6J) cell line and the LY2 (BALB/c) cell line, with either control or EphB4 shRNA knockdown [18]. RT was incorporated in these studies as it is a key component of HNSCC treatment [24, 25] and enhances local control to allow time for evaluation of distant metastases. Concordant with our previous findings [18], downregulation of EphB4 in cancer cells increases local tumor growth in both models (Supplementary Fig. 1A–F). For metastasis studies, MOC2 cells were implanted in the buccal mucosa while LY2 cells were implanted in the floor of the mouth. Tumors were irradiated and imaged with computed tomography (CT) scans to monitor disease progression (Fig. 1A, D, Supplementary Fig. 1G). Interestingly, EphB4 knockdown in cancer cells was associated with an increase in the incidence of lung (MOC2, Fig. 1B, C) or mediastinal lymph node (LY2, Fig. 1E, F) metastases. Histological validation of the metastatic lesions was performed based on hematoxylin and eosin (H&E) staining of lung tissues (Supplementary Fig. 1H). These findings indicate that EphB4 plays a multifaceted role in tumor biology, and that its downregulation in cancer cells increases not only local progression, but also distant spread.

### Loss of EphB4 in cancer cells induces protein dysregulation concomitant with increased metastatic capacity

**Fig. 2:**
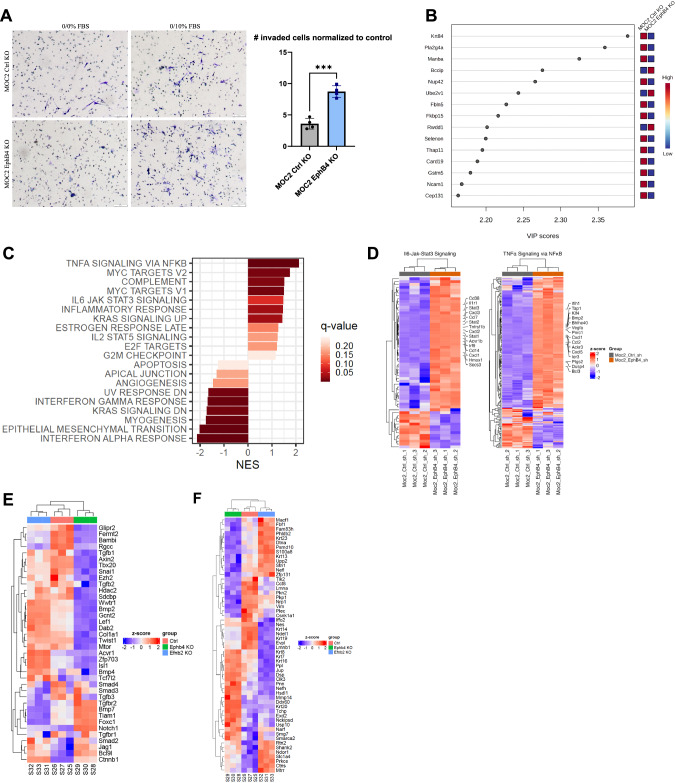
Loss of EphB4 in cancer cells induces protein dysregulation concomitant with increased metastatic capacity. **A** Representative images and quantification for Boyden chamber invasion assay conducted on MOC2 control (*n* = 4) or EphB4 (*n* = 4) CRISPR knockout (KO) cell lines. **B** Variable importance in projection (VIP) score plots of mass spectrometry proteomics data conducted on MOC2 EphB4 KO (*n* = 5) and Ctrl (*n* = 5) tumors displaying upregulation of BCCIP and Ube2v1 proteins in EphB4 KO tumors compared to control tumors. **C** Hallmark pathways generated from RNA-sequencing of MOC2 control (*n* = 3) versus EphB4 (*n* = 3) shRNA cell lines. **D** RNA-sequencing of MOC2 control (*n* = 3) and EphB4 (*n* = 3) shRNA knockdown (KD) cells showing expression of genes associated with IL6-Jak-Stat3 and TNF-alpha signaling via NFκB. **E** Expression of genes quantified using RNA-sequencing of MOC2 control (*n* = 3), EphB4 (*n* = 3), and ephrinB2 (EFNB2) (*n* = 3) CRISPR KO cells. **F** Expression of genes associated with intermediate filament cytoskeleton quantified using RNA-sequencing of MOC2 control (*n* = 3), EphB4 (*n* = 3), and ephrinB2 (*n* = 3) CRISPR knockout cell lines. The experiments were performed once with their own biological replicates. For Boyden chamber quantification, comparison of invaded cells between the control and experimental group was done using a two-sided student’s t-test. Significance was determined if the *p*-value was < 0.05*, < 0.01**, < 0.001***, and < 0.0001****. *p*-values are indicated for the figures (**A**) ***p = 0.0002. The error bars represent the standard error of the mean ( ± SEM).

To investigate whether EphB4 loss enhances cancer cell migration, we employed Transwell Boyden chamber assays. We observed that EphB4 knockout cancer cells had increased migratory capacity compared to control cells in vitro (Fig. 2A). Mass spectrometry proteomics analysis also revealed signatures consistent with enhanced metastatic potential in EphB4 knockout cells (Fig. 2B). The variable importance in projection (VIP) score plots derived from mass spectrometry-based proteomics data comparing MOC2 EphB4 knockout tumors to control tumors unveiled a significant upregulation of BCCIP and Ube2v1 proteins (Fig. 2B). This finding holds substantial clinical relevance because overexpression of BCCIP has been associated with a poor prognosis and implicated in the facilitation of proliferation and migration in lung adenocarcinoma [26]. In addition, Ube2v1, a member of the ubiquitin conjugating E2 enzyme variant proteins, plays a pivotal role in promoting EMT and metastasis by epigenetically suppressing autophagy at the transcriptional level [27]. The observed upregulation of BCCIP and Ube2v1 in EphB4 knockout tumors suggests their potential involvement in tumorigenesis and metastatic progression.

To investigate whether EphB4 enhances metastasis through a canonical cancer-cell centered mechanism, we explored intrinsic changes induced by loss of EphB4 in the cancer cell using RNA sequencing. We also observed an increase in metastasis-related hallmark pathways including TNF-alpha signaling via NF-κB, MYC, IL6/JAK/STAT3 signaling, and KRAS in MOC2 EphB4 knockdown cells (Fig. 2C) [28–32]. Notably, TNF-alpha signaling via NF-κB and IL6/JAK/STAT3 signaling have been widely characterized to foster an inflammatory tumor microenvironment that favors metastasis [33, 34]. Delving deeper into genes associated with TNF-alpha signaling via NF-κB and IL6/JAK/STAT3 signaling, we observed upregulation of chemokines CXCL1, CXCL2, CXCL3, CXCL5, CCL2, and CCL7, which have been shown to promote metastasis through enhanced cellular proliferation, migration, and invasion (Fig. 2D) [35–38]. Comparison of transcriptomic changes between MOC2 control and EphB4 knockout cell lines also showed higher expression of TGF beta receptors 1 and 2, NOTCH1, and FOXC1, a transcription factor implicated in cancer cell plasticity, treatment resistance, invasion, and epithelial-mesenchymal transition (EMT) (Fig. 2E) [39, 40]. Surprisingly, the EMT hallmark pathway was significantly lower in MOC2 EphB4 knockdown cells compared to controls (Fig. 2C). To investigate whether the observed increase in metastasis upon loss of EphB4 is driven by non-classical EMT, we used mass spectrometry proteomics analysis of MOC2 EphB4 knockout cell lines and tumors. While E-cadherin downregulation and vimentin upregulation are hallmarks of classical EMT [41], we observed opposite trends upon knockout of cancer cell EphB4 in vitro and in vivo (Supplementary Fig. 2A, B). Our RNA sequencing data also show upregulation of Snai1 and Twist1 along with downregulation of vimentin in EphB4 knockout cells compared to controls (Fig. 2E, F). We also observed upregulation of Wnt signaling components WNT5a and WNT7a, non-canonical proteins that regulate cell functions such as proliferation, motility, and migration (Supplementary Fig. 2C) [42]. Together, these data suggest that cancer cell EphB4 likely regulates metastasis through non-canonical EMT and Wnt signaling.

Additionally, comparison of EphB4 knockout versus control cells revealed significant alterations in genes associated with the cytoskeleton (Fig. 2F). Notably, EphB4 knockout cancer cells had low expression of Nefl, which has been shown to be associated with nodal spread and poor prognosis in breast cancer patients (Fig. 2F) [43]. Nefl also induces apoptosis, suppresses growth, and decreases invasion of HNSCC cancer cells, offering potential mechanisms by which Nefl downregulation in EphB4 knockout cancer cells enhances their metastatic capacity [44]. Additionally, EphB4 knockout cells had increased expression of matrix metallopeptidase 14, pinin, and keratins 7, 8, 16, and 20 (Fig. 2F), genes associated with increased proliferation, and metastasis across multiple cancer types [45–50]. Collectively, these results provide an association between the absence of EphB4 and cancer cell polarization towards a pro-metastatic phenotype, as EphB4 knockout induces molecular changes in MOC2 cancer cells that affect regulation of the cytoskeleton, migration, and non-classical EMT. These changes may contribute to tumor progression and metastasis, highlighting the role of cancer cell EphB4 in regulating metastasis.

### Knockdown of EphB4 in cancer cells combined with radiation treatment promotes suppressive intratumoral immune populations

**Fig. 3:**
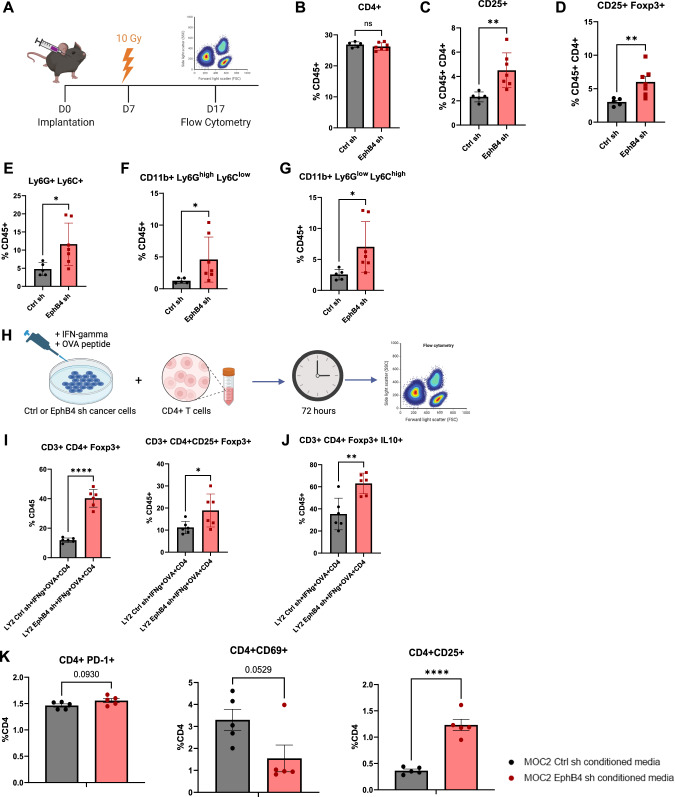
Knockdown of EphB4 in cancer cells combined with radiation treatment promotes suppressive intratumoral immune populations. **A** Experimental design for C57BL/6 J mice implanted with MOC2 cancer cells. The buccal tumor received single dose of radiation treatment (10 Gy) on day 7 after implantation. **B**–**G** Flow cytometric quantification of CD4 + T cells (**B**), regulatory T cells (Tregs) defined by CD4 + CD25+ (**C**) and CD4 + CD25+ Foxp3+ expression (**D**), myeloid-derived suppressor cells (MDSCs) defined by CD45 + Ly6G + Ly6C+ (**E**), CD45 + CD11b+ Ly6G^high^ Ly6C^low^ (**F**), and CD45 + CD11b+ Ly6G^low^ Ly6C^high^ (**G**) in the tumor microenvironment (TME) (Ctrl sh, *n* = 5; EphB4 sh *n* = 7). **H** Experimental design for coculture of LY2 Ctrl or EphB4 shRNA cancer cells with CD4 + T cells. Cancer cells were incubated with interferon-gamma (IFNg) for 48 h, then OVA peptide overnight. CD4 + T cells from DO11.10 BALB/c mice were then cocultured with the cancer cells for 72 hours and subsequently harvested for flow cytometry. **I** Flow cytometry quantification of Tregs defined by CD4+ Foxp3+ and CD4 + CD25+ Foxp3+ expression (*n* = 6 per group). **J** Flow cytometry quantification of Treg immunosuppressive function defined by IL-10 expression (*n* = 6 per group). **K** Flow cytometry quantification of CD4 + T cells treated with conditioned media from MOC2 control or EphB4 shRNA cells showing PD-1, CD69, and CD25 expression (*n* = 5 per group). The experiments were performed once with their own biological replicates. Comparison between control and experimental groups was done using a Mann Whitney test for the in vivo experiment and a two-sided student’s *t*-test for the in vitro experiments. Significance was determined if the *p*-value was < 0.05*, < 0.01**, < 0.001***, and < 0.0001****. *p*-values are indicated for the figures (**B**) ns *p* = 0.3636, (**C**) **p = 0.0025, (**D**) **p = 0.0051, (**E**) *p = 0.0177, (**F**) *p = 0.0101, (**G**) *p = 0.0101, (**I**) CD3 + CD44+Foxp3+ ****p < 0.0001; CD3 + CD4 + CD25+Foxp3+ *p = 0.0394, (**J**) **p = 0.0027, (**K**) ****p < 0.0001. The error bars represent the standard error of the mean ( ± SEM).

Building upon our RNAseq and mass spectrometry proteomics data (Fig. 2), we used flow cytometry analysis to further understand the implications of cancer cell EphB4 knockdown in vivo (Fig. 3A). Although the overall frequency of total CD4 + T cells was the same in both EphB4 knockdown and control groups, we observed that EphB4 knockdown led to an increase in intratumoral Tregs (Fig. 3B–D). We also observed upregulated markers associated with myeloid-derived suppressor cells (MDSCs) such as Ly6C + Ly6G+ (Fig. 3E), CD11b+Ly6G^high^Ly6G^low^ granulocytic MDSCs (Fig. 3F), and CD11b+Ly6G^low^Ly6C^high^ monocytic MDSCs (Fig. 3G). To determine if EphB4 knockdown in the cancer cell directly affects the CD4 + T cell state, we cocultured control or EphB4 shRNA cancer cells with CD4 + T cells and conducted flow cytometry (Fig. 3H, Supplementary Fig. 3). Our findings revealed that EphB4 knockdown in cancer cells polarizes CD4 + T cells towards the Treg phenotype (Fig. 3I) and enhances Treg immunosuppression (Fig. 3J). These findings complement our previous work, which established increased VEGF upon knockdown of cancer cell EphB4 in vitro and in vivo [18]. VEGF contributes to the establishment of a pro-tumorigenic immune microenvironment through recruitment of Tregs and MDSCs, polarization of macrophages towards an M2 phenotype, inhibition of dendritic cell maturation, and upregulation of checkpoint inhibitors on effector T cells [51, 52].

Given the observed increase in Tregs with EphB4 knockdown in the cancer cell, we investigated whether CD4 + T cells within the TME differentiate into Tregs by treating CD4 + T cells with conditioned medium from MOC2 EphB4 knockdown cancer cells in vitro and conducting flow cytometry. We found a trend towards higher PD-1 expression in CD4 + T cells treated with EphB4 knockdown conditioned medium, indicative of greater exhaustion [53] (Fig. 3K). EphB4 knockdown conditioned medium also yielded a trend towards decreased CD4 + T cell activation (defined by CD69 expression) and a significant increase in CD25+ Tregs (Fig. 3K). These data are consistent with our previous work showing that EphB4 knockdown in cancer cells increases intratumoral IL-10 and G-CSF, thereby promoting Treg differentiation and immunosuppression [18].

In order to elucidate the underlying mechanism by which EphB4 shRNA influences Treg function, Tregs isolated from the spleen and lymph nodes of tumor-bearing mice (MOC2 control shRNA or EphB4 shRNA) were sorted and subjected to mass spectrometry proteomics analysis (Supplementary Fig. 4A). Gene ontology (GO) processes revealed a concurrent upregulation of actin polymerization and depolymerization processes, leukocyte migration, and inflammatory response (Supplementary Fig. 4B). Intriguingly, EphB4 shRNA-mediated knockdown also led to downregulation of pathways related to T helper 1 (Th1) and T helper 2 (Th2) cell differentiation as well as the mTOR signaling pathway, as indicated by KEGG pathway analysis (Supplementary Fig. 4C). Remarkable decreases were also observed in microtubule organizing center function and TOR signaling by GO pathway analysis (Supplementary Fig. 4D). Notably, the observed downregulation in mTOR signaling may contribute to Treg stabilization and maintenance of an immunosuppressive TME through the PI3K-Akt-mTOR signaling [54]. These findings shed light on the potential molecular pathways through which EphB4 downregulation modulates Treg function.

Our data show that loss of cancer cell EphB4 results in a combination of immunosuppression and intrinsic changes in tumor cell behavior, thereby allowing for proliferation of the primary tumor and dissemmination of cancer cells to distant sites [55]. This provides valuable insights into therapeutic targets that could be used for regulating immune responses in the context of HNSCC. Furthermore, EphB4-driven modulation of the anti-tumor immune response likely synergizes with changes in cancer cell metastatic capacity (Fig. 2) to promote distant metastasis.

### Knockdown of ephrinB2 in the vasculature significantly reduces distant metastases

**Fig. 4:**
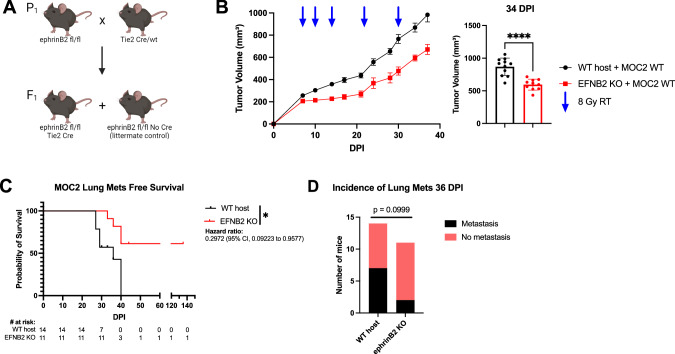
Knockout of ephrinB2 in the vasculature significantly reduces distant metastases. **A** Breeding strategy for creating mice with ephrinB2 (EFNB2) KO on vascular endothelial cells. **B** Average tumor volume curve and dot plot showing significantly smaller average tumor volume at 34 DPI in ephrinB2^fl/fl^Tie2^Cre^ (EFNB2 KO) mice (*n* = 10) implanted with MOC2 tumors compared to wild-type (WT) hosts (*n* = 11). **C** Kaplan-Meier curves showing lung metastasis free survival of WT hosts (*n* = 14) and ephrinB2 KO (*n* = 11) mice. Numbers at risk indicate mice that were alive without metastases at specified timepoints. **D** Contingency table showing incidence of lung metastases detected by CT scans in ephrinB2 KO mice (*n* = 11) versus WT hosts (*n* = 14) by 36 DPI. The experiment was replicated twice. Comparison of tumor volume between the control and experimental group was done using a two-sided student’s *t*-test. For Kaplan-Meier survival curves, significance was determined by a log-rank Mantel-Cox test. For contingency tables indicating the incidence of metastases, significance was determined by a Chi-square test. Significance was determined if the *p*-value was < 0.05*, < 0.01**, < 0.001***, and < 0.0001****. *p*-values are indicated for the figures (**B**) ****p < 0.0001, (**C**) *p = 0.0172. The error bars represent the standard error of the mean ( ± SEM).

Since EphB4’s ligand ephrinB2 is expressed in blood vessels [56, 57], we sought to determine how vascular ephrinB2 affects metastatic spread of HNSCCs. Using the ephrinB2^fl/flTie2Cre^ (ephrinB2 KO) mouse model (Fig. 4A) [18] implanted with MOC2 cancer cells, we found that deletion of vascular ephrinB2 coupled with RT significantly reduced local tumor growth compared to wild-type (WT) hosts (Fig. 4B, Supplementary Fig. 5). EphrinB2 knockout in vascular endothelial cells also reduced the incidence of lung metastasis, leading to significantly increased lung metastasis-free survival (Fig. 4C, D). Additionally, our cell line RNA sequencing data in MOC2 ephrinB2 knockout cells compared to controls showed lower expression of EMT-related genes such as Smad3, Smad4, TGFb3, TGFBR2, BMP7, Tiam1, Foxc1, Fermt2, Bambi, Notch1, and TGFBR1 (Fig. 2E, F) which are typically associated with enhanced migratory and invasive capacities of cancer cells [58–62]. Conversely, there is an increase in Acvr1, Zfp703, Isl1, and Tcf712 (Fig. 2E), suggesting that ephrinB2 loss shifts the tumor microenvironment (TME) toward a less invasive phenotype [63–65]. These findings collectively underscore the multicompartmental contributions of ephrinB2 in modulating HNSCC metastasis, highlighting it as a potential therapeutic target.

### Knockout of ephrinB2 in vascular endothelial cells enhances anti-tumor immune cell populations in the TME

**Fig. 5:**
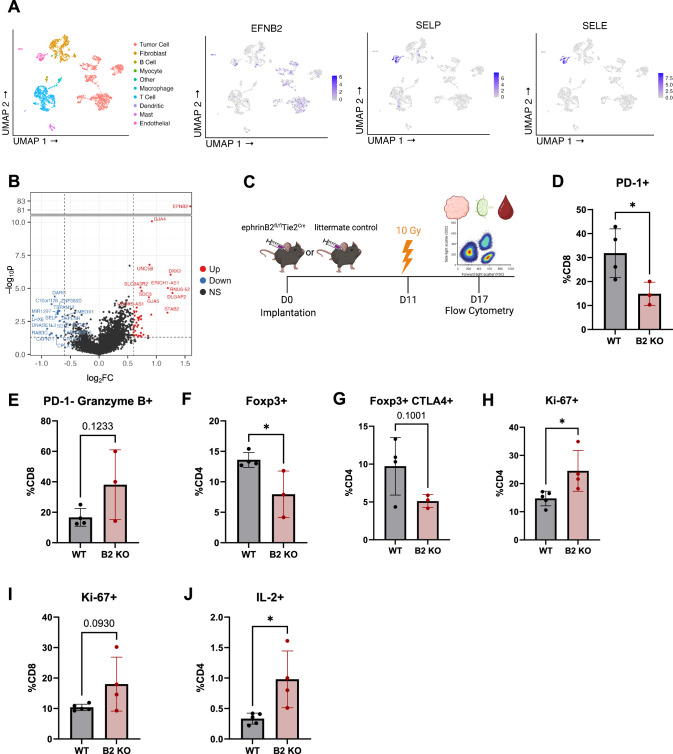
Knockout of ephrinB2 in vascular endothelial cells enhances anti-tumor immune cell populations in the TME. **A** UMAP plots showing expression of ephrinB2, SELP, and SELE in endothelial cells from human HNSCC single-cell sequencing. **B** Volcano plot showing differential gene expression of endothelial cells that do (red) or do not (blue) express ephrinB2 from human HNSCC single-cell sequencing. **C** Experimental design for flow cytometric analysis of MOC2 WT tumors implanted in ephrinB2 KO (B2 KO) mice and littermate controls. **D**–**G** Flow cytometry analysis displaying quantification of PD-1 expressing CD8 + T cells (**D**), PD-1^-^ Granzyme B^+^ expressing CD8 + T cells (**E**), Tregs defined by Foxp3 expression (**F**), and immunosuppressive Tregs defined by the addition of CTLA-4 expression (**G**) in the TME (WT, *n* = 4; B2 KO, *n* = 3). **H**–**J** Flow cytometry analysis of proliferating CD4 + T cells defined by Ki-67 expression (**H**), proliferating CD8 + T cells defined by Ki-67 expression (**I**), and IL-2 expressing CD4 + T cells (**J**) in the draining lymph nodes (DLNs) (WT, *n* = 5; B2 KO, *n* = 4). The experiments were performed once with their own biological replicates. Comparison of differences between the control and experimental group was done using a two-sided student’s *t*-test. Significance was determined if the *p*-value was < 0.05*, < 0.01**, < 0.001***, and < 0.0001****. *p*-values are indicated for the figures (**D**) *p = 0.0466, (**F**) *p = 0.0358, (**H**) *p = 0.0247, (**J**) *p = 0.0177. The error bars represent the standard error of the mean ( ± SEM).

To investigate how vascular ephrinB2 shapes the TME in HNSCC, we analyzed a publicly available scRNA-seq dataset of HNSCC patients [66]. We first confirmed expression of ephrinB2, SELP, and SELE in endothelial cells (Fig. 5A). Next, gene expression profiles of endothelial cells derived from patients with high ephrinB2 expression were compared to those without ephrinB2 expression. Notably, these analyses show that endothelial cells with high ephrinB2 expression have lower levels of SELP and SELE, both of which are genes associated with promoting transendothelial migration of effector T cells (Fig. 5B) [67].

To further validate that vascular ephrinB2 modulates the immune cell composition of the TME after RT, we analyzed RNA sequencing of vascular endothelial cells isolated from C57BL/6 mice subjected to total body irradiation (5 Gy x 1) (GSE141790). Six hours after RT, vascular endothelial cells were found to upregulate ephrinB2 RNA expression (Supplementary Fig. 6A). EphrinB2 upregulation after RT was also confirmed in our MOC2 HNSCC tumor model, and ephrinB2-expressing vascular cells were observed to be more proliferative as determined by Ki-67 expression (Supplementary Fig. 6B, C). These data suggest that RT upregulates ephrinB2 in the vasculature, which may allow selective infiltration of immune cells.

We then conducted flow cytometry to examine how vascular ephrinB2 affects intratumoral immune cell composition in the context of RT (Fig. 5C, Supplementary Fig. 7). Within the TME, we found decreased PD-1 expression in CD8 + T cells, indicative of decreased exhaustion [68], along with increased granzyme B expression (Fig. 5D, E). There was a significant decrease in Tregs and a trend towards decreased Treg expression of CTLA-4, suggesting decreased Treg-mediated immunosuppression [69] (Fig. 5F, G).

Given the observed differences in the primary tumors, we sought to determine whether the changes seen in immune cells were limited to the TME due to tumor-targeted RT or whether global knockout of vascular ephrinB2 induced systemic changes. Analysis of the immune response in the blood and draining lymph nodes (DLNs) was conducted to answer this question (Supplementary Fig. 8A, C). A trend towards higher CD8 + T cell granzyme B production was also observed in the circulation (Supplementary Fig. 8B). Within the DLNs, there was increased proliferation of CD4+ and CD8 + T cells (Fig. 5H, I), although no differences were found in their percentages (Supplementary Fig. 8D, E). We also observed significantly higher CD4 + T cell activation and a Th1 phenotype, identified by increased IL-2 production (Fig. 5J). Additionally, DLNs of ephrinB2 KO mice showed a trend towards increased dendritic cells (DCs), suggesting an environment where T cell priming may be more likely to occur (Supplementary Fig. 8F). Similar to the TME, we observed a trend towards fewer Tregs in the DLNs (Supplementary Fig. 8G). Collectively, these data suggest that vascular ephrinB2 may facilitate distant metastasis at least in part by dampening the systemic immune response.

To evaluate the effects of targeting ephrinB2 on lymphocyte extravasation into the TME, we conducted specialized flow cytometry experiments involving adoptive transfers of CD4 + T cells into tumor-bearing mice (Supplementary Fig. 9A) [70, 71]. Specialized flow cytometry was conducted to quantify extravasation by distinguishing between intravascular and intratumoral lymphocytes (Supplementary Fig. 9A) [70–72]. This enabled us to extrapolate transendothelial migration from ratios of CD4 + T cells in the tumor versus in the vessels. Optimization studies were performed to determine that 1 hour following adoptive transfer was the appropriate time to allow CD4 + T cell extravasation within the MOC2 TME, as this timepoint preceded a rise in intratumoral CD4 + T cells (Supplementary Fig. 9A, B). We found higher ratios of vessels to tumor CD4+ cells in ephrinB2 knockout mice, suggesting increased CD4 + T cell accumulation in the TME (Supplementary Fig. 9C). Collectively, our data support inhibition of vascular ephrinB2 to enhance infiltration of anti-tumor immune populations and combat cancer progression.

### EphrinB2-Fc-His and Fc-TNYL-RAW-GS reduce local and distant metastasis in C57BL/6 mice implanted with MOC2 cells

Since our current and previous data [18, 19]) suggest that it would be therapeutically desirable to maintain or activate EphB4 signaling in tumor cells while also inhibiting ephrinB2 reverse signaling in the vasculature, we examined the in vivo effects of a dimeric ephrinB2-Fc fusion protein in our MOC2 mouse model of metastasis. Ephrin Fc fusion proteins bind to the ephrin-binding pocket in the ligand-binding domain of Eph receptors to activate receptor forward signaling, inhibit Eph receptor-ephrin interaction, and thus inhibit ephrin reverse signaling [73]. We also examined the effects of Fc-TNYL-RAW-GS, a version of the EphB4-targeting TNYL-RAW peptide dimerized by fusion of its N-terminus to Fc, since N-terminal dimerization has been shown to transform TNYL-RAW from an antagonist to an agonist [74, 75]. The TNYL-RAW peptide, like ephrinB2-Fc, targets the ephrin-binding pocket of EphB4 [75] and inhibits ephrinB2 reverse signaling [76]. However, TNYL-RAW is more specific since it only binds to EphB4 among the Eph receptors [75], whereas ephrinB2 also binds to the other EphB receptors and to EphA4 [77].

**Fig. 6:**
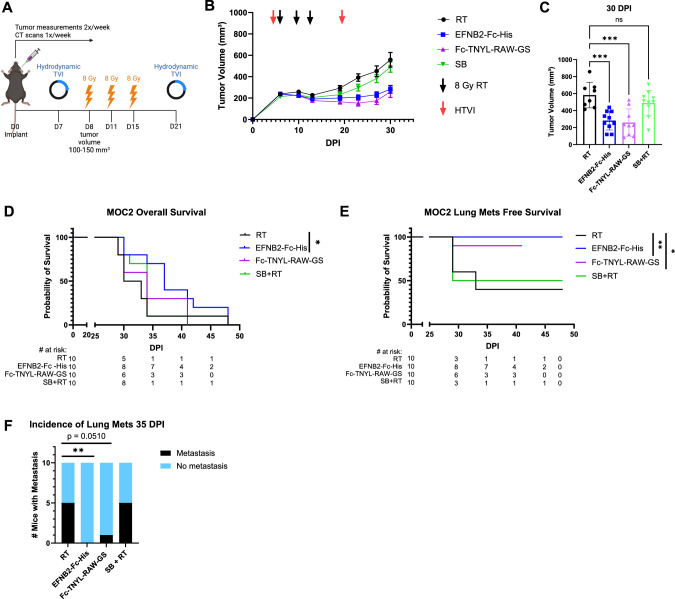
EphrinB2-Fc-His and Fc-TNYL-RAW-GS reduce local tumor growth and distant metastasis in C57BL/6 mice implanted with MOC2 cells. **A** Experimental design for C57BL/6 J mice implanted with MOC2 WT cancer cells and treated with different plasmids (*n* = 10 per group). Hydrodynamic tail vein injections (HTVI) and radiation therapy (8 Gy) were administered as indicated. Mice treated with RT alone or Sleeping Beauty (SB) + RT served as controls. **B** Average tumor volume curves comparing effects of different plasmids on local tumor growth. **C** Dot plot showing the effects of different plasmids on tumor volume 30 DPI. **D**, **E** Kaplan-Meier curves showing therapeutic effects of different plasmids on overall survival (**D**) and lung metastasis free survival (**E**) in MOC2 WT implanted C57BL/6 mice. For Kaplan-Meier curves showing lung metastasis free survival, numbers at risk indicate mice that were alive without metastases at specified timepoints. **F** Contingency table quantifying the incidence of lung metastasis detected by CT scans in MOC2 WT implanted mice by 35 DPI. The experiment was replicated twice. Comparison of tumor volume between the control and experimental groups was done using a Dunnett post hoc test after one-way ANOVA was performed. For Kaplan-Meier survival curves, significance was determined by a log-rank Mantel-Cox test. For contingency tables indicating the incidence of metastases, significance was determined by a Chi-square test. Significance was determined if the *p*-value was < 0.05*, < 0.01**, < 0.001***, and < 0.0001****. *p*-values are indicated for the figures (**C**) RT vs EFNB2-Fc-His ***p = 0.0003; RT vs Fc-TNYL-RAW-GS ***p = 0.0002; RT vs SB + RT ns *p* = 0.3757, (**D**) *p = 0.0485, (**E**) RT vs EFNB2-Fc-His **p = 0.0075; RT vs Fc-TNYL-RAW-GS *p = 0.0488, **F** **p = 0.0098. The error bars represent the standard error of the mean ( ± SEM).

We used hydrodynamic injection of plasmids encoding ephrinB2-Fc-His (EFNB2-Fc-His) or Fc-TNYL-RAW-GS to transfect mouse liver cells and the Sleeping Beauty (SB) system to achieve prolonged protein expression and secretion of the Fc proteins by liver cells into the mouse circulation (Fig. 6A) [78, 79]. Administration of ephrinB2-Fc-His or Fc-TNYL-RAW-GS in conjunction with RT reduced local tumor growth and improved overall mouse survival (Fig. 6B–D, Supplementary Fig. 10). Additionally, ephrinB2-Fc-His and Fc-TNYL-RAW-GS increased lung metastasis-free survival compared to controls (Fig. 6E). Notably, by the time half of the control mice had lung metastases detected by CT scans, mice treated with ephrinB2-Fc-His had no incidence of metastases (Fig. 6F).

### EphrinB2-Fc-His activates EphB4 without concurrent activation ephrinB2 while Fc-TNYL-RAW-GS activates both EphB4 and ephrinB2

**Fig. 7:**
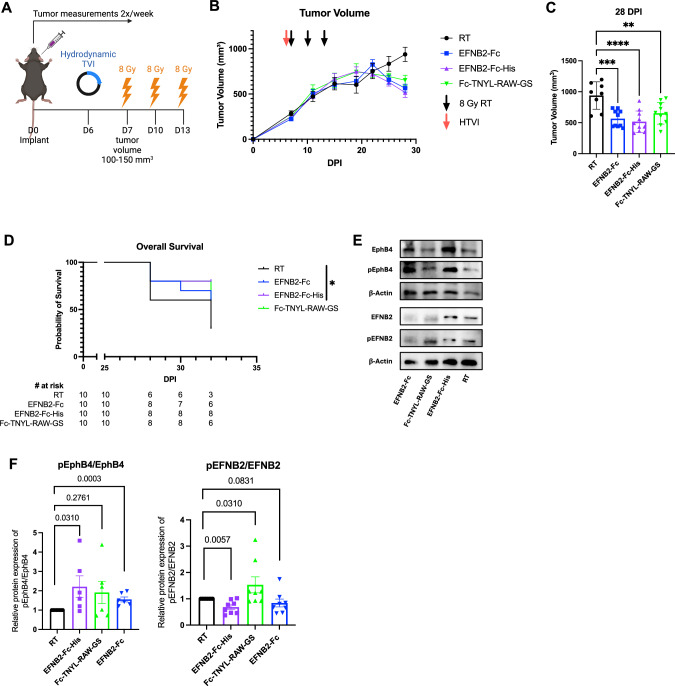
EphrinB2-Fc-His activates EphB4 without concurrent activation of ephrinB2 while Fc-TNYL-RAW-GS activates both EphB4 and ephrinB2. **A** Experimental design for C57BL/6 J mice implanted with MOC2 WT cancer cells. Hydrodynamic tail vein injections (HTVI) and radiation therapy (8 Gy) were administered as indicated (*n* = 10 per group). Mice treated with RT alone served as controls. **B** Average tumor volume curve comparing effects of different plasmids on local tumor growth. **C** Dot plot showing the effects of different plasmids on tumor volume 28 DPI. **D** Kaplan-Meier curves showing therapeutic effects of different plasmids on overall survival in MOC2 WT implanted C57BL/6 mice. **E** Western blots showing protein expression of EphB4, phospho-EphB4 (pEphB4), ephrinB2 (EFNB2), phospho-ephrinB2 (pEFNB2), and beta-actin in control and experimental tumors. **F** Dot plots showing ratios of protein expression of pEphB4/EphB4 (*n* = 6 per group) and pEFNB2/EFNB2 (*n* = 8 per group) in control and experimental tumors. Protein bands were quantified using Image lab and ImageJ software. The experiments were performed once with their own biological replicates. For the Kaplan-Meier overall survival curves, significance was determined by a log-rank Mantel-Cox test. Comparison of tumor volume between the control and experimental groups was done using a Dunnett post hoc test after one-way ANOVA was performed. Comparison of relative protein expression between the control and experimental groups was done using Mann Whitney tests. Significance was determined if the *p*-value was < 0.05*, < 0.01**, < 0.001***, and < 0.0001****. *p*-values are indicated for the figures (**C**) RT vs EphrinB2-Fc ***p = 0.0002; RT vs EFNB2-Fc-His ****p < 0.0001; RT vs Fc-TNYL-RAW-GS **p = 0.0044, (**D**) *p = 0.0389. The error bars represent the standard error of the mean ( ± SEM).

To validate the mechanisms through which ephrinB2-Fc-His and Fc-TNYL-RAW-GS reduce local tumor growth and the formation of distant metastases, we repeated hydrodynamic injections of these plasmids and included ephrinB2-Fc (EFNB2-Fc) for comparison (Fig. 7A). Concordant with our previous data, ephrinB2-Fc, ephrinB2-Fc-His, and Fc-TNYL-RAW-GS all significantly reduced local tumor growth, while ephrinB2-Fc-His was the only treatment that yielded a significant improvement in overall survival (Fig. 7B–D). Tumors were harvested and processed for western blotting, then probed for expression of EphB4, phospho-EphB4 (pEphB4), ephrinB2 (EFNB2), and phospho-ephrinB2 (pEFNB2) (Fig. 7E). Comparing the ratios of relative protein expression of pEphB4/EphB4 between control and experimental tumors, ephrinB2-Fc and ephrinB2-Fc-His significantly activated EphB4 while Fc-TNYL-RAW-GS trended towards EphB4 activation (Fig. 7F). Additionally, the ratios of relative protein expression of pEFNB2/EFNB2 in control and experimental tumors demonstrated that ephrinB2-Fc-His significantly reduces activation of ephrinB2 while Fc-TNYL-RAW-GS significantly activates ephrinB2 (Fig. 7F).

To verify successful in vivo expression of Fc fusion proteins, serum was collected 26 days following hydrodynamic injection and probed by immunoblotting with anti-human Fc antibodies. Fc fusion proteins were detected in the serum of mice injected with ephrinB2-Fc-His and Fc-TNYL-RAW-GS (Supplementary Fig. 11A). Additionally, an ELISA was performed to measure EphB4 phosphorylation in PC3 prostate cancer cells and EphB4 stably expressing HEK293 cells following treatment with commercially available ephrinB2-Fc (ephrinB2-Fc R&D), ephrinB2-Fc-His, and Fc-TNYL-RAW-GS (Supplementary Fig. 11B). Compared to the Fc control, all three treatments resulted in phosphorylation of EphB4 (Supplementary Fig. 11B).

Our data suggest that EphB4 acts as a tumor suppressor by inhibiting both the growth of primary tumors and the formation of metastases. In contrast, vascular ephrinB2 acts as a promoter of local tumor growth and distant metastases. Our phopho-western blot data support that activating EphB4 while simultaneously avoiding activation of ephrinB2 using various therapeutic strategies such as the ephrinB2-Fc-His plasmid provides maximal benefit to reducing local tumor growth and distant metastases. Taken together, our results emphasize the role of EphB4 as a suppressor of cancer cells’ intrinsic metastatic capacity, which can be amplified by agents that activate EphB4. Our data also underscore the therapeutic benefit of inhibiting ephrinB2 signaling in vascular endothelial cells and systemically. For the first time, we also provide therapeutic approaches that can activate EphB4 and inhibit ephrinB2, therefore serving as key translational strategies.

## Discussion

The EphB4-ephrinB2 signaling axis is aberrantly expressed in a variety of cancers, highlighting it as a promising therapeutic target [14, 15, 80–82]. While some studies implicate EphB4-ephrinB2 signaling as a pro-metastatic driver [16, 82], others show the opposite [15, 83]. Contributing to the complexity is the fact that EphB4 and ephrinB2 can have independent, dichotomous effects on primary tumor growth and metastasis [17, 18, 21, 84]. Consequently, it is increasingly apparent that context is key to deciphering the role of this receptor-ligand pair, both in terms of cancer type and cellular compartment [9, 85]. However, many studies on EphB4-ephrinB2 signaling in HNSCC metastasis have been limited to descriptive correlations and/or heavy reliance on in vitro experiments [20, 21, 23, 86]. Our study provides novel insight into the intricate dynamics of EphB4-ephrinB2 signaling in HNSCC metastasis in vitro and in vivo.

Specifically, we illuminate the role of EphB4 knockdown in cancer cells, shedding light on its impact on metastatic potential and the immune microenvironment. Our findings indicate that knockdown of EphB4 in cancer cells leads to increased metastasis in two models of HPV-unrelated HNSCC due to multiple factors. Our proteomic and RNAseq data indicate that the aggressive phenotypes of MOC2 EphB4 knockdown or knockout may be mediated, at least in part, through non-classical (hybrid) EMT mechanisms or alternative signaling pathways. We observed downregulation of key classical EMT transcription factors Snai1, Twist1, and vimentin [87, 88] and upregulation of E-cadherin protein expression, all of which are features that are uncommon in classical EMT [89]. Additionally, the upregulation of WNT5a, WNT7a, Tiam1, and Foxc1 suggest that loss of EphB4 may activate alternative signaling pathways that enhance cell motility, invasion, and metastatic potential [90–92]. Thus, EphB4 loss may shift the balance of signaling and structural components to favor tumor proliferation and metastasis through non-classical EMT pathways that likely involve enhanced Wnt signaling and ECM remodeling.

Our mass spectrometry proteomics analysis of EphB4 knockdown in MOC2 cells revealed dysregulation of proteins involved in cell division, which may enhance the intrinsic capacity of cancer cells to metastasize. Our RNA sequencing data also show significant alterations in immune and tumor-related pathways, including downregulation of Th1/Th2 cell differentiation and mTOR signaling. These findings suggest that EphB4 expressed in cancer cells modulates Treg function. Notably, we observed an augmentation in the prevalence of immunosuppressive Tregs and myeloid cells in vivo, potentially contributing to tumor immune evasion and providing insights into cancer immunotherapy targets within the TME. These data suggest that this increase in intratumoral Tregs is driven by two processes – direct conversion of CD4 + T cells and increased infiltration of Tregs into the tumor.

Our lab and others have identified the vasculature as a main compartment of ephrinB2 activity in multiple cancer types including HNSCC [16, 18, 93]. We previously reported that ephrinB2 knockout in the vascular endothelium in the absence of RT decreases local tumor growth and the abundance of Tregs in the TME [18]. In this study, we sought to examine vascular ephrinB2 from a metastatic angle and incorporated RT due to the ability of RT to enhance anti-tumor immunity [94–99] and its use as a standard-of-care therapy for HNSCC patients [24, 25]. Our characterizations of ephrinB2-expressing endothelial cells and extravasation data suggest that vascular ephrinB2 may modulate trafficking of lymphocytes through regulation of adhesion molecules. We observed that RT coupled with deletion of vascular ephrinB2 decreases metastasis in a preclinical mouse model of HNSCC. Additionally, our data show that vascular ephrinB2 loss combined with RT enhanced systemic CD4+ and CD8 + T cell activation, trended towards decreased Treg immunosuppression, and reduced CD4 + T cell entry into the tumor. As a result, concurrent RT and inhibition of vascular ephrinB2 is a potential therapeutic avenue to mitigate both local and distant HNSCC progression.

While this work primarily focuses on vascular ephrinB2’s modulation of the immune microenvironment, we did not rule out the possibility that loss of ephrinB2 on vessel endothelial cells may also reduce metastasis by preventing cancer cell intravasation. However, our previous work established improved vessel perfusion and vascular normalization with combined knockout of ephrinB2 on HNSCC cells and vascular endothelial cells [18]. Loss of ephrinB2 could strengthen the vascular barrier, potentially limiting tumor cells’ ability to invade and enter the bloodstream [100]. Supporting this, our RNAseq data show decreased expression of pro-migratory and pro-invasive genes, such as Fermt2 and Tiam1, in ephrinB2 knockout MOC2 cancer cells (Fig. 2E). Fermt2 and Tiam1 are implicated in cellular migration and invasion [101–104], suggesting that disruption of ephrinB2 signaling impairs mechanisms that facilitate tumor cell penetration of vessel walls. Additionally, enhanced anti-tumor immune activity, including increased CD8 + T cell responses and reduced Tregs (Fig. 5B–G) [68, 69], may further limit tumor cells’ access to the vasculature. While we cannot conclusively rule out tumor intravasation as a mechanism of reduced metastasis, our findings strongly suggest that both impaired tumor cell invasion and enhanced immune activity may contribute to reduced metastatic potential.

The promiscuous and compensatory nature of Eph receptors and ephrins [9, 77, 105] poses a challenge in efforts to specifically target metastasis-promoting components while maintaining protective signaling components. Our findings show that EphB4 and ephrinB2 have opposing effects on metastasis in the HPV-unrelated HNSCC MOC2 and LY2 models. Additionally, our data suggest that, in contrast to EphB4, genetic deletion of ephrinB2 in the vasculature enhances both local and distant tumor control (Fig. 4B–D). Consistently, we observe that ephrinB2-Fc-His treatment, which activates pEphB4 and inhibits pEFNB2, leads to a reduction in both local tumor progression and metastasis (Fig. 7B–D). However, activation of both pEphB4 and pEFNB2 with Fc-TNYL-RAW-GS fails to impart similar efficacy on tumor control or distant metastasis. Moreover, our data from genetically engineered mouse models demonstrate that ephrinB2 knockout on the vessel downregulates the transcription of key EMT-related genes such as Smad3, Smad4, TGFb3, TGFBR2, BMP7, Tiam1, Foxc1, Fermt2, Bambi, Notch1, and TGFBR1 (Fig. 2E). These are genes known to enhance tumor invasiveness and migration [58–62, 106]. This suggests that ephrinB2 inhibition may reduce EMT and promote a less invasive tumor phenotype. This is consistent with prior studies and the existing literature that ephrinB2 inhibition can shift cancer cells towards an epithelial phenotype [107–109].

Clinical trials incorporating broad receptor pan-targeted tyrosine kinase inhibitors that may affect Eph-ephrin signaling, including erlotinib, dasatinib, and cetuximab, have largely failed to provide meaningful clinical benefit due to limited efficacy and resistance [110–113]. Given the structural similarities and signaling cascades (PI3K, mTOR, MAPK, JAK/STAT, etc.) shared among receptor tyrosine kinases [114, 115], inhibitors targeting them also pose the risk of off-target effects, including potential inhibition of the Eph receptor and ephrin gene families. Consequently, exploration of more targeted therapeutics is needed to improve patient response.

A notable finding in this study is the capacity of ephrinB2-Fc-His and Fc-TNYL-RAW-GS to decrease local tumor growth and the incidence of lung metastases in a preclinical model of HNSCC. Given their high specificity for their targets (EphB receptors and EphA4 for ephrinB2-Fc-His or EphB4 for Fc-TNYL-RAW-GS), the two Fc fusion plasmids are promising agents to combat HNSCC metastasis and circumvent off-target effects of traditional receptor tyrosine kinase inhibitors [74–76, 116]. The benefits observed in our preclinical models open the door to further exploration of other Fc fusion proteins targeting Eph receptors and ephrins depending on their expression levels and presumed effects in certain cancer types. In light of our findings, one potential therapy to abrogate HNSCC metastasis would be an EphB4 agonist that targets the ephrin-binding pocket to activate EphB4 and inhibit ephrinB2.

## Materials and Methods

### Cell culture and reagents

Murine HNSCC tumor cell lines MOC2 and LY2 were used for all in vivo studies. The MOC2 cell line was obtained from Ravindra Uppaluri (Dana-Farber Cancer Institute) and is derived from a C57BL/6 mouse with squamous cell carcinoma of the oral cavity that was exposed to DMBA for 25 weeks. The LY2 cell line was obtained from Dr. Nadarajah Vigneswaran (University of Texas Health Science Center). The LY2 cell line was isolated from lymph node metastases that developed in BALB/c mice after inoculation of PAM 212 squamous cell carcinoma cells [117]. All cell lines were cultured at 37 °C and 5% CO_2_. The LY2 cell lines were cultured in DMEM-F12 media supplemented with 10% fetal bovine serum, 2% Primocin, and 1% Fungin (InvivoGen, San Diego, California, USA). The MOC2 cell line was cultured in DMEM-F12:IMDM (2:1) supplemented with 2% Primocin and 1% Fungin (InvivoGen, San Diego, California, USA), 1.75 ng EGF, 20 ng hydrocortisone per 500 mL of media, and 0.1% insulin (Sigma Aldrich, St. Louis, Missouri, USA). HEK293AD human embryonic cells (#240085, Agilent, Santa Clara, California, USA) and PC3 prostate cancer cells (#CRL-1435, ATCC) were cultured in Dulbecco’s Modified Eagle Medium (DMEM; #10-013-CV, Corning, Corning, New York, USA) and RPMI 1640 medium(#11875093, ThermoFisher Scientific, Waltham, Massachusetts, USA), supplemented with 10% fetal bovine serum and 1% antimycotics and antibiotics (#A5955, Sigma-Aldrich, St. Louis, Missouri, USA). HEK293AD cells were stably transfected with pLVX-IRES-Neo encoding full-length human EphB4 with an N-terminal FLAG tag using Lipofectamine 2000 reagent according to the manufacturer’s instruction (#11668019, ThermoFisher Scientific). Forty-eight hours after transfection, the cells were selected with 1 mg/ml G418 (#10131035, ThermoFisher Scientific) for 15 days to generate stably transfected cells. T cells were cultured in RPMI media supplemented with recombinant human IL-2 (Biological Resources Branch National Cancer Institute, Frederick, Maryland, USA), 10% fetal bovine serum, 50 nM 2-mercaptoethanol, 1X MEM non-essential amino acids, 1X penicillin-streptomycin, 1 M HEPES, and 100 mM sodium pyruvate (Gibco, Billings, Montana, USA).

### Fc fusion proteins

Commercial purified mouse ephrinB2-Fc was purchased from R&D Systems (#496-EB-200, which contains a C-terminal His tag). The ephrinB2-Fc-His plasmid was obtained by cloning the cDNA encoding the mouse ephrinB2 extracellular region (residues 3-227), a GSGDP linker, the Fc portion of human IgG1, a glycine, and a His tag into the PT3-EF1a-C-Myc plasmid. The Fc-TNYL-RAW-GS plasmid was obtained by cloning a cDNA encoding the CD5 signal peptide followed by the Fc portion of human IgG1 and the sequence ARTNYLFSPNGPIARAWGS, with the underlined sequence representing the TNYL-RAW peptide [75]. The Fc control plasmid was obtained by cloning a cDNA encoding the CD5 signal peptide followed by the Fc portion of human IgG1. The PT3-EF1a-C-Myc and pCMV/SB11 plasmids were kindly provided by Dr. Gen-Sheng Feng (University of California, San Diego) [118].

Purified ephrinB2-Fc-His and Fc-TNYL-RAW-GS proteins for cell stimulation were generated by transiently transfecting HEK293 cells with the respective plasmids and the pCMV/SB11 plasmid at a 1:10 ratio. Cells were passaged into a larger plate the next day. Upon reaching 70% confluence, the culture medium was replaced with fresh Opti-MEM (#31985088, ThermoFisher Scientific) after washing the cells twice with warm DPBS (#21-030-CV, Corning). After three days, the culture medium was collected, and HEPES, pH 7.5 was added to a final concentration of 10 mM before centrifugation at 1000 g for 10 min to remove cell debris. The resulting supernatant was incubated with GammaBind Plus Sepharose beads (#17088602, Cytiva, Marlborough, Massachusetts, USA) overnight at 4 °C. Beads were washed with cold PBS once before bound Fc fusion proteins were eluted with 0.1 M glycine HCL, pH 2.5, and the low pH in the eluates was neutralized with 1 M Tris HCl buffer, pH 7.5.

### Animal tumor models

For studies using only wild-type hosts, 5- to 7-week-old female C57BL/6 mice were purchased from the Jax Labs, Bar Harbor, Maine, USA. For studies using ephrinB2^fl/fl^Tie2^Cre^ mice (C57BL/6 background), breeding pairs were obtained from Dr. Mohit Kapoor’s lab (University Health Network, University of Toronto, Canada) and maintained at the Anschutz Medical Campus, Aurora mouse facility. Deletion of ephrinB2 from the vasculature of ephrinB2^fl/fl^Tie2^Cre^ mice was done as previously described [18]. Experiments were performed once enough ephrinB2^fl/fl^Tie2^Cre^ were bred, and 5- to 10-week-old female mice were used and age matched with controls. In vivo orthotopic HNSCC tumor models were established as previously described [96, 119]. For buccal tumor implantations, 1 × 10^5^ MOC2 cells per 50ul of serum-free cell media were prepared. For floor of mouth implantations, 1 × 10^5^ LY2 cells per 50 uL of serum-free cell media were prepared. A 1:1 mixture of cells and Matrigel (10 mg/mL, BD Biosciences, San Jose, California, USA) at a volume of 100ul was injected into the right buccal mucosa of the mice (MOC2) or floor of mouth (LY2). Mice were appropriately age matched and randomized into groups, with treatment beginning when tumors reached ~100–200 mm^3^ (MOC2) or ~150–300 mm^3^ (LY2). Tumor size was measured twice weekly using digital calipers, and tumor volume was estimated using the equation V = AxB2/2, where A is the longest diameter of the tumor and B is the shortest. Computed tomography scans were performed once a week as described below. Mice were euthanized when the mice reached study end qualifications approved by the Institutional Animal Care and Use Committee (IACUC). Mice were euthanized under CO_2_, and intracardiac blood collection was performed. Blood was placed in serum collection tubes (BD, Franklin Lakes, New Jersey, USA) and centrifuged at 6000 rpm for 2 min to collect the serum supernatant. Tumors were harvested and flash frozen in liquid nitrogen, while lung tissues were harvested and placed in 10% formalin for further processing. Both sera and tumors samples were stored in -80°C for subsequent analysis.

For studies using ephrinB2-Fc and Fc-TNYL-RAW-GS plasmids, C57BL/6 J mice were initially implanted with 100k MOC2 cancer cells. 7 days later, mice were randomized into different groups including a control group treated with radiation therapy alone, a control group transfected with 1 µg of Sleeping Beauty transposase plasmid (SB), and experimental groups transfected with 1 µg of SB in addition to 20 µg of EFNB2-Fc or Fc-TNYL-RAW-GS plasmid. Sleeping Beauty transposase plasmid was used to promote cDNA integration into mouse liver genomic DNA. Liver transfection was performed by hydrodynamic tail vein injection as described previously [19]. A repeat liver transfection was performed 3 weeks post-tumor implantation.

All in vivo experiments were performed using a sample size of *n* = 3-20 mice per group unless noted otherwise. Power analysis was used to decide on the number of mice per group. For effect studies, no mice were excluded from the data shown. Investigators were blinded during group allocation and analysis for the experiments included in the study. All animal protocols used in this study were approved by IACUC of the University of Colorado, Denver.

### Irradiation and computed tomography scans

Mice were anesthetized using isoflurane before and during the procedure. The mice were then placed in an X-RAD image guided small animal irradiator (Precision X-Ray, Bradford, Connecticut, USA). For tumor irradiation, the irradiation field was determined using fluoroscopy for each mouse. After the field was established, the mice were irradiated with 225kVp/20 mA with a copper filter, which corresponds to 5.6 Gy/min for 98 s (8 Gy) or 123 s (10 Gy). For computed tomography scans, mice were scanned using an aluminum filter. For MOC2 metastasis studies, 3D lung contouring was generated using ITK-Snap. For flow studies, mice received one fraction of 10 Gy. For MOC2 metastasis studies, three fractions of 8 Gy were administered to the buccal tumor. For LY2 floor of mouth studies, three fractions of 8 Gy were administered to the tumor. In some studies utilizing the MOC2 cell line, tumors were given an additional dose of 8 Gy RT as they approached 500 mm^3^ in volume to reduce local tumor burden and prolong the study. For transendothelial migration studies, mice received one dose of 10 Gy 12 to 14 days after implantation.

### Flow cytometry

Tumors and draining lymph nodes (DLNs) were harvested from mice and immediately put on ice in Hank’s Balanced Salt Solution (HBSS). To facilitate single cell suspension of lymphocytes from tumors, the tumors were minced and then incubated for 30 min at 37 °C with 200U of Collagenase III (Worthington, Lakewood, New Jersey, USA). Both tumors and draining lymph nodes were then filtered through 70 µm nylon filters. The tumors were then spun down at 400 g for 5 min and resuspended in 3 mL of Red Blood Cell Lysis Buffer for 3 min. After 3 min, 6 mL of HBSS was added and all the samples were spun down again at 400 g for 5 min. Blood was taken using an intracardiac puncture. The blood was spun down for 10 min at 400 g before resuspending in 3 mL of Red Blood Cell Lysis Buffer for 3 min. After 3 min, 6 mL of HNSCC was added to the sample and the samples were spun down at 400 g for 5 min. The samples were aspirated and then were plated in 24 well plates in 1 mL of RPMI with 0.1% monensin to prevent cytokine release by the golgi apparatus and 0.2% brefeldin A with cell stim cocktail of PMA and ionomycin to simulate cytokine production and transport in the cells. The cells were incubated in the stim media for 4 hours at 37 °C. The plates were then spun down and the samples were resuspended in 200 µl of Fc Block (anti-CD16/CD32 antibody, Tonbo Biosciences, San Diego, California, USA) for 20 min at room temperature. The samples were then plated into a 96 well plate and spun down at 400 g for 5 min. The samples were then resuspended in 100 µl of PBS containing 5 µl of live/dead aqua viability stain (Invitrogen, Carlsbad, California, USA). The samples were incubated away from light for 15 min at room temperature. The samples were then spun down and resuspended in a mixture of the extracellular antibodies in brilliant stain buffer (BD Biosciences, Franklin Lakes, New Jersey, USA). The cells were incubated for 30 min at room temperature. After incubated the cells were washed twice with FA3 buffer. The cells were then incubated overnight with the Foxp3 transcription factor staining kit (eBioscience, San Diego, California, USA). The next day the cells were spun down and washed twice as per the kit instructions. The cells were then stained with the intracellular antibodies in brilliant stain buffer for 30 min at room temperature. The cells were then washed twice with PBS. Cells were re-suspended and then run on an Aurora Spectral Flow Cytometer (Cytek Biosciences, Fremont, California, USA) at the Barbara Davis Center Diabetes Research Center Cell and Tissue Analysis Core. For all flow cytometry data, if too few events were available for analysis ( < 10 cells), the sample was either not included in the analysis or cell counts were shown for each sample.

The following antibodies were used in these studies: PerCP-CD45 (Clone: 30-F11 Biolegend, San Diego, California, USA, 557235), PerCP-Cy5.5- CD3 (Clone: 17A2 Biolegend, 100217), BUV805-CD3 (Clone: 17A2 BD Biosciences, 741982), SB436-CD4 (Clone: GK1.5 eBioscience, 17-0041-82), eF450-CD4 (Clone: GK1.5 eBioscience, 48-0041-82), BUV496-CD4 (Clone: GK1.5 BD Biosciences, 612952), BB515-CD8 (Clone: 53-6.7 BD Biosciences, 564422), BV570-CD44 (Clone: IM7 Biolegend, 103037), AF532-Foxp3 (Clone: FJK-16s eBioscience, 58-5773-82), eF660-CD11b (Clone: M1/70 Invitrogen, 50-112-4253), BUV661-CD11b (Clone: M1/70 BD Biosciences, 612977), BV711-CD25 (Clone: PC61, Biolegend 102049), NovaFluor Yellow 590-CD11b (Clone: ICRF44, eBioscience, H036T03Y02-A), eF450-LY6C (Clone; HK1.4, eBioscience, 48-5932-82), BV650-LY6C (Clone: HK1.4 Biolegend, 128049), BV570-LY6C (Clone: HK1.4 Biolegend, 128029), BV605-LY6C (Clone: HK1.4 Biolegend, 128035), PerCP Cy5.5-LY6G (Clone: 1A8 Biolegend, 127615), BV421-LY6G (Clone: 1A8 Biolegend, 127627), BV650-LY6G (Clone: 1A8 BD Biosciences, 740554), BV605-LY6G (Clone: 1A8 Biolegend, 127639), AF594-ephrinB2 (1° Catalogue number AF496 R&D; 2° Ref A21468), BV480-F4/80 (Clone: T45-2342 BD Horizon, 565635), AF-647-IL2 (Clone: JES6-5H4, Biolegend 503814), BV605-IL-2 (Clone: JES6-5H4 Biolegend, 503829), PE-IL-2 (Clone: JES6-5H4 Biolegend, 503807), BV421-IL-10 (Clone: JES5-16E3 Biolegend 505021), BUV395-PD-1 (Clone: J43 BD Biosciences, 744549), APC-R700-CTLA-4 (Clone: UC10-4F10-11 BD Biosciences, 565778), APC-Fire 750-Granzyme B (Clone: QA16A02 Biolegend, 372209), APC-iNOS (Clone: CXNFT eBioscience, 17-5920-82), PerCP-eFlour 710-Arginase 1 (Clone: A1exF5 eBioscience, 46-3697-82), APC-eFluor 780-Ki-67 (Clone: SoIA15, Invitrogen 47-5698-82), BV480-Ki-67 (Clone: B56 BD Biosciences, 566109), BV786-Ki-67 (Clone: B56 BD Biosciences, 563756), BUV805-MHCII (Clone: M5/114.15.2 BD Biosciences, 748844), PE-Dazzle 594-MHCII (Clone: M5/114.15.2 Biolegend, 107648), PE-Cy7-CD31 (Clone: 390 Biolegend, 102418), SB436-PDPN (Clone: eBio.1.1(8.1.1) Invitrogen, 62-5381-82), Live Dead Aqua (Invitrogen, L34965).

The following antibodies were used in intravital flow studies: PerCP-CD45 (Clone: 30-F11 Biolegend, 557235), PerCP-Cy5.5- CD3 (Clone: 17A2 Biolegend, 100217), eF450-CD4 (Clone: GK1.5 Invitrogen, 48-0041-82), AF532-Foxp3 (Clone: FJK-16s Invitrogen, 58-5773-82), Live Dead Aqua (Invitrogen, L34965).

### Human HNSCC Single-Cell RNA sequencing analysis

Single cell RNA-Seq data [66] was downloaded from the UCSC Cell Browser [120] as an expression matrix, metadata, and UMAP coordinates. R software (v4.2.3) was used with package Seurat75 (v4.3.0) to visualize cell populations and import UMAP coordinates and metadata. EphrinB2, SELE, and SELP expression were assessed on cells phenotyped as endothelial cells in the UCSC-provided metadata, and TPM values followed a bimodal distribution where cells could be assumed as ephrinB2 low and high expressors. Log2-transformed fold changes and student’s t-test were calculated to create a volcano plot comparing endothelial cells with high ephrinB2 expression versus low ephrinB2 expression.

### T cell isolation and ex vivo activation

CD4+ and regulatory T cells were isolated from dissociated spleens using a CD4 + CD25+ regulatory T cell isolation kit according to manufacturer’s instructions (Miltenyi Biotec, Bergisch Gladbach, North Rhine-Westphalia, Germany). CD4 + T cells were activated in a 24-well plate (Corning, New York, USA) coated with 2 μg/mL anti-CD3 (clone 2C11, Invitrogen, Waltham, Massachusetts) and 2 μg/mL anti-CD28 (clone PV-1, BioXCell, Lebanon, New Hampshire, USA). Two days after activation, CD4 + T cells were removed from the coated plate and split to a concentration of 1 × 10^6^ cells/mL. Subsequent splits were performed as needed. 30 U/mL recombinant human IL-2 (Biological Resources Branch National Cancer Institute) was added on day 0 and every other day of activation.

### T cell fluorescent dye-labeling

7 to 10 days following ex vivo activation, T cells were stained with carboxy-fluorescein diacetate succinimidyl ester (CFSE), CellTrace Yellow, or CellTrace Violet proliferation dyes (Invitrogen, Waltham, Massachusetts, USA) at a 1:1000 ratio in serum-free RPMI for 15–20 min at 37 °C. Excess stain was quenched by incubation with 1X volume FBS at room temperature for 2 min, then cells were washed twice with PBS prior to use.

### Intravascular staining

Staining of lymphocytes in the vasculature was done by intravascular staining as previously described [71]. APC-conjugated anti-CD4 antibody (clone GK1.5, Biolegend, San Diego, California, USA, 100412) was administered by tail vein injection 3 min prior to CO2 euthanasia. After euthanizing, perfusions were performed by injection of PBS through the heart to wash out excess stain.

### Transendothelial migration flow

C57BL/6 and ephrinB2^fl/fl^Tie2^Cre^ mice were implanted with 100k MOC2 control or EphB4 shRNA tumors and irradiated 12 to 14 days post-implantation as described above. Two days later, ex vivo activated CD4 + T cells were fluorescently stained with CFSE, and 1 × 10^7^ cells were adoptively transferred by tail vein injection. 30 min, 60 min, 90 min, 2 h, 6 h, 12 h, and 24 h following adoptive transfer, intravascular staining and perfusions were conducted as described above. Tumors were then harvested and processed for flow cytometry as described above. Optimization studies demonstrated that 1 h was the appropriate time for capturing T cell accumulation in this tumor model. Extravasation flow was therefore performed 1 h after adoptive transfers for subsequent experiments.

### RNA-sequencing analysis

Sample preparation and RNA-sequencing analysis were conducted as described previously [18, 97].

### Boyden chamber invasion assay

MOC2 control or EphB4 shRNA cells were plated to 70% confluency, then the media was replaced with a 1:1 ratio of complete media and Fc-containing conditioned media and the cells were incubated overnight. Cells were then serum starved and 24-well plate inserts with 8 µM pores were coated with a 1:10 ratio of Matrigel (Corning) and serum-free media for 4 h in a 37 °C incubator. Following incubation, 750 µL complete media containing 10% FBS was added to the bottom chamber, then 500k serum-starved cells were seeded in the Matrigel-coated top chambers. For controls, 750 µL of media containing 0% FBS was added to the bottom chamber. Cells were incubated in a 37°C incubator and allowed to migrate for 24 h. Next, cells were fixed in 3.7% formaldehyde diluted in PBS for 2 min at room temperature followed by 2 washes in PBS. The cells were permeabilized in 100% methanol for 20 min, washed twice with PBS, then stained with 0.1% crystal violet for 15 min at room temperature. Non-invasive cells on the top of the insert were scraped off with cotton swabs, then the inserts were allowed to dry. Inserts were imaged at 10x magnification, and the number of migrated cells was counted using ImageJ (National Institutes of Health, Bethesda, Maryland, USA). The number of invaded cells in each experimental condition was normalized to the number of invaded cells in the control groups where serum-free media was added to the bottom chamber. Each condition was performed in five replicates.

### Cancer cell and CD4 + T cell coculture assay

LY2 control or EphB4 shRNA cancer cells were initially cultured in complete DMEM/F12 medium described above. Subsequently, 100,000 cells were plated overnight in 6-well plates. Cells were then treated with 10 ng/ml of IFN-gamma for 48 h, after which 10 µg/ml of OVA peptide was added overnight. CD4 + T cells were then isolated from the spleen and lymph nodes of BALB/c mice (DO11.10) using a CD4 + T cell isolation kit according to the manufacturer’s instructions (Miltenyi Biotec, Bergisch Gladbach, North Rhine-Westphalia, Germany). These isolated CD4 + T cells were activated as described above before being co-cultured with LY2 cells at a ratio of 1:1 for 72 h. Finally, the cells were harvested and stained for flow cytometric analysis.

### Whole-cell lysate preparation

Tumor tissues from the indicated group were homogenized in RIPA buffer (Millipore) with a protease inhibitor cocktail (Thermo Fisher Scientific) and phosphatase inhibitors (Sigma-Aldrich) on ice for 30 min. Lysates were then collected, and protein concentration was measured using a standard BCA assay as previously described [121].

### Immunoblot

For western blotting of tumor tissue, proteins were denatured at 95 °C for 7 min and then stored at −20 °C. Equal amounts of protein (15 μg) were separated using 10% sodium dodecyl sulfate–polyacrylamide (SDS) gels and transferred onto PVDF membranes (#10600023, Amersham Biosciences, Piscataway, New Jersey, USA). To block nonspecific binding sites, the membranes were incubated with 5% Bovine Serum Albumin in Tris-buffered saline containing 1% Tween for 1 h at room temperature. The membranes were then probed with the specified primary antibodies. Blots were probed overnight at 4 °C with respective antibodies. Primary antibodies anti-EFNB2 (#131536, Abcam, Cambridge, United Kingdom), anti-pEFNB2- pTyr316 (SAB 4300631, Sigma), anti-EphB4 (#37-1800, Invitrogen), anti-pEphB4-Tyr987 (PA5-64792, Invitrogen) and anti-β-actin -HRP conjugate (#5125, Cell Signaling Technology, Danvers, Massachusetts, USA). Horseradish peroxidase (HRP)-conjugated secondary antibodies were obtained from Sigma. Membranes were washed thrice and the antibodies visualized with enhanced chemiluminescent HRP substrate (#R-03031-D25 and R-03025-D25, Advansta, San Jose, California, USA). For detection of signals, X-ray films or versa doc were used. To confirm loading control after detection of phosphorylated protein, membranes were stripped using ReBlot Plus strong antibody stripping solution (#2504, Merck, Germany), blocked and re-probed with different antibodies against whole protein. Protein bands were quantified using *Image lab* & *ImageJ* software. Results are shown as the ratio of total protein or phospho-protein to B actin normalized to the control group. To assign the right protein size, Precision Plus Protein Dual Color Standards Protein Marker was used as a marker (6 μl, #1610374, BIO-RAD).

For immunoblotting of Fc fusion proteins in vivo, mouse serum and purified human IgG Fc (#0855911, MP Biomedicals, Irvine, California, USA) were diluted in LDS sample buffer (#B0007, Life Technologies, Carlsbad, California, USA) with 2.5% β-mercaptoethanol, heated at 95 °C for 2 min, and run on Bolt Bis-Tris Plus gels (#NW04125Box, ThermoFisher Scientific). After semi-dry transfer, the Immobilon membranes were blocked with 5% BSA in 0.1% Tween 20 in Tris buffered saline (TBS) for 1 h and then incubated in the cold overnight with an anti-human IgG antibody at a 1:1000 dilution (#109-005-098, Jackson ImmunoResearch Labs, West Grove, Pennsylvania, USA). Blots were washed 3 times with 0.1% Tween 20 in TBS before incubating with a horseradish peroxidase-conjugated anti-goat secondary antibody at a 1:4000 dilution (#A16005, Life Technologies) for 1 h. After 3 washes, the chemiluminescence signal was captured using the ChemiDoc Touch Imaging System (Bio-Rad, Hercules, California, USA). Chemidoc images were quantified using Image Lab (Bio-Rad) to determine the approximate concentration of Fc proteins in the mouse serum using known concentrations of human Fc as standards.

### ELISA

HEK293AD cells stably expressing EphB4 plated on poly-D-Lysine coated plates, or PC3 cells plated without coating, were cultured to 70–80% confluency. Cells were serum starved for 1 h before treating with ephrinB2-Fc, Fc-TNYL-RAW, or Fc for 30 min. Cells were rinsed once with cold DPBS (#21-030-CV, Corning), and collected with lysis buffer (0.5% TX-100 in PBS with Halt Protease and Phosphatase Inhibitor Cocktail (#78443, ThermoFisher Scientific)). After 15 min of incubation on ice, cell lysates were centrifuged at 16,000 g at 4 °C for 10 min, and supernatants were collected. The supernatants were used with the Human Phospho-EphB4 DuoSet IC ELISA kit (#DYC4057-2, R&D Systems, Minneapolis, Minnesota, USA) according to the manufacturer’s instructions.

### Hematoxylin and eosin staining and imaging

Lung samples were submitted to the Dermatology Histology Core at CU Anschutz for cutting and hematoxylin and eosin staining.

### Statistical analysis

Statistical tests were chosen appropriate to sample sizes and the number of group comparisons. A student’s t-test or one or two-way ANOVA was used when comparing groups for tumor growth curves. A log-rank Mantel-Cox test was used to determine significance in survival studies. The Dunnett post hoc test was used after one-way ANOVA where multiple experimental groups were involved. For flow analysis, a student’s t-test or Mann Whitney test was used to compare control and experimental groups. For western blot analysis, a Mann Whitney test was performed to compare relative protein expression between control and experimental groups. A Chi-square test was used to compare incidence of metastasis between groups. All statistical analysis was done in Prism Software (v9.1.0). Significance was determined by *p*-values: *p < 0.05, **p < 0.01, ***p < 0.001, ****p < 0.0001.

## Supplementary information

Supplemental Figures

Supplemental Figure Legends

## Data Availability

The human HNSCC single-cell RNA-seq dataset [66] used in this study are publicly available data in the Gene Expression Omnibus database under the accession code GSE103322. The remaining data and datasets generated and/or analyzed in this study are available from the corresponding author on reasonable request.
