# Supplementary material for: Manipulating the EphB4-ephrinB2 axis to reduce metastasis in HNSCC
Source: Oncogene. 2024 Nov 3;44(3):130–46. doi: 10.1038/s41388-024-03208-9 (PMC11725500; doi:10.1038/s41388-024-03208-9)

**Supplemental Figure 1: Loss of EphB4 in cancer cells significantly increases local tumor growth in the context of radiotherapy.**

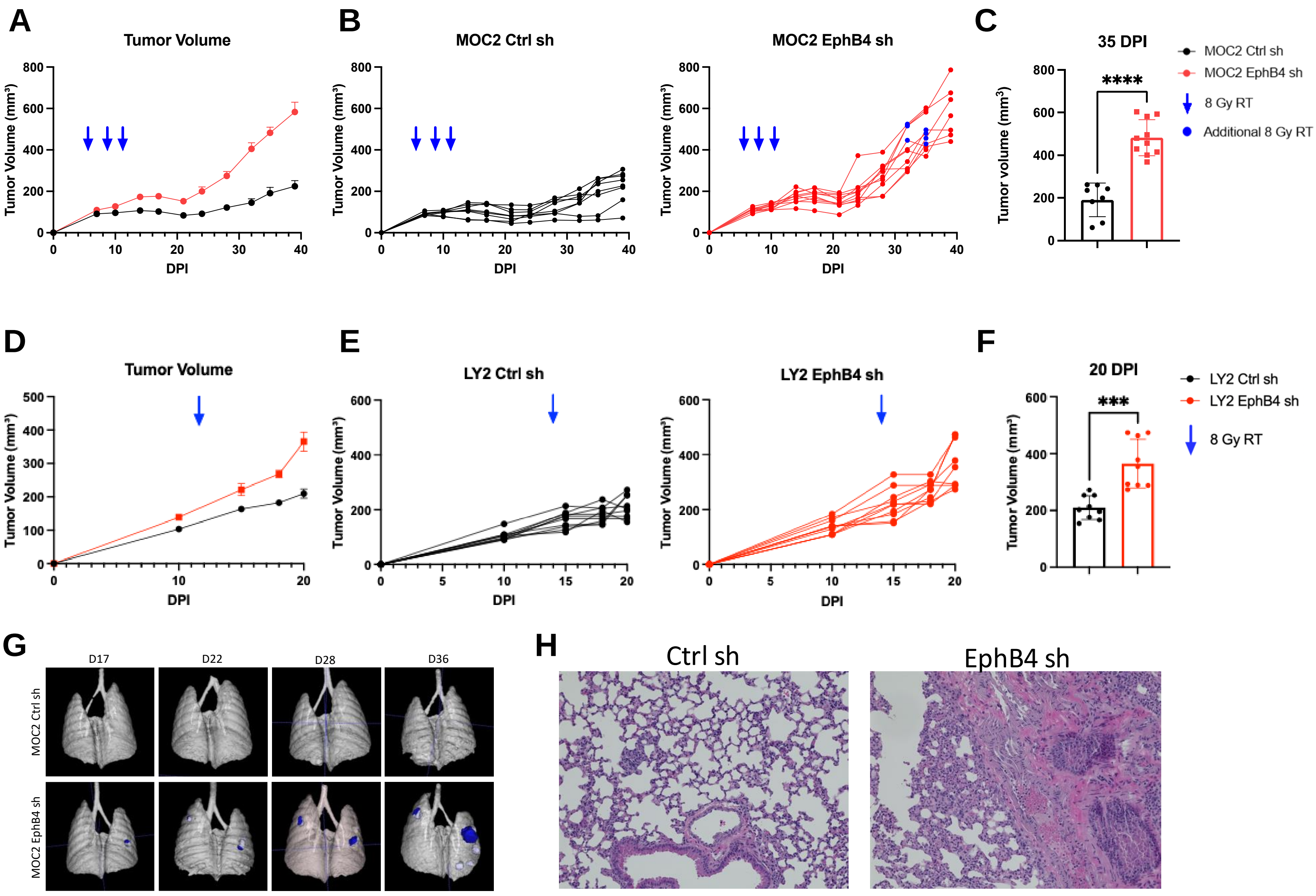

**Supplemental Figure 2: EphB4 KO cancer cells express increased E-cadherin, Wnt-5a, and Wnt7a as well as decreased vimentin *in vitro* and *in vivo*.**

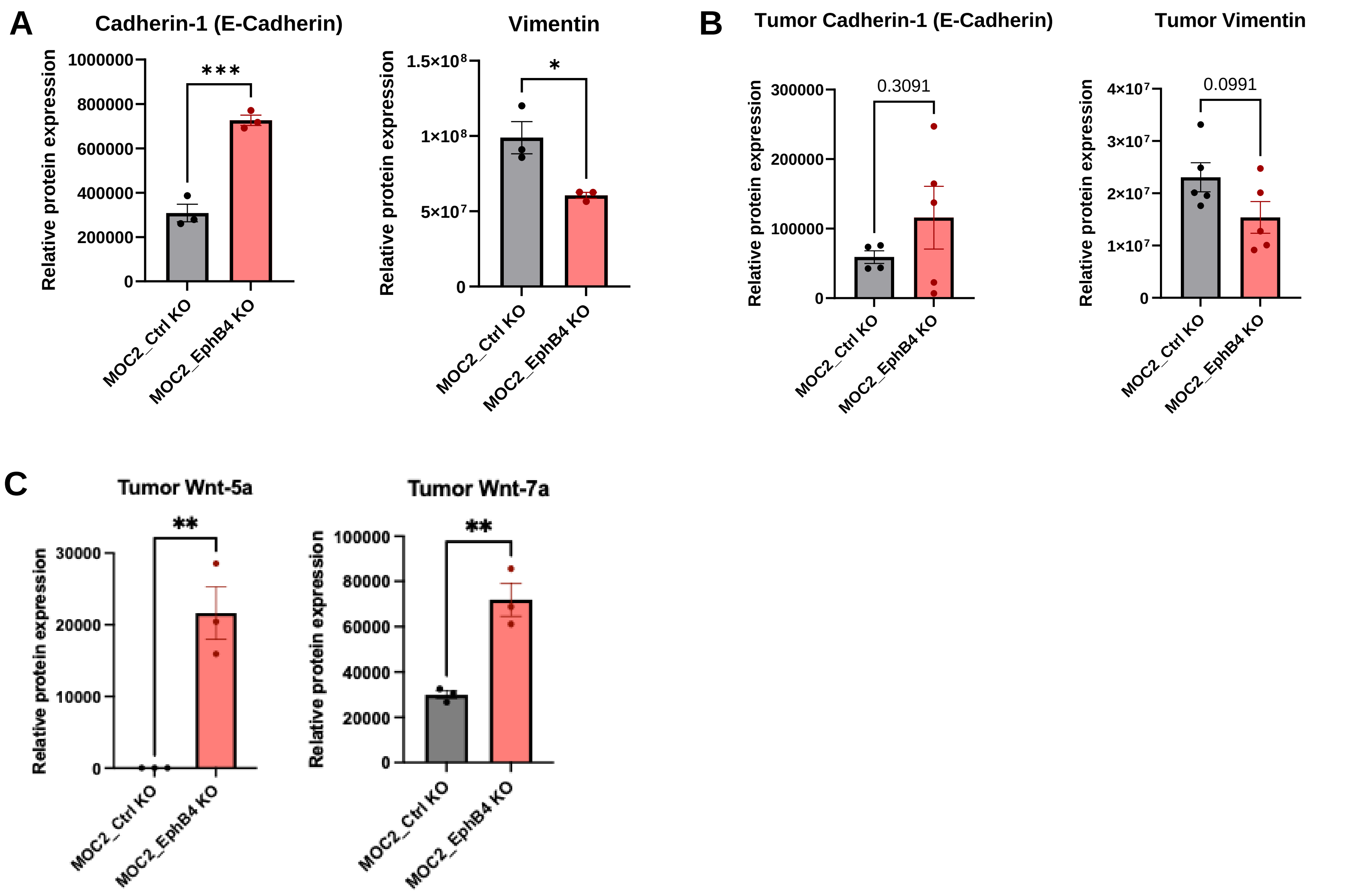

**Supplemental Figure 3: Gating strategy for cancer cell and CD4+ T cell coculture experiment**

**A** **Gating strategy**

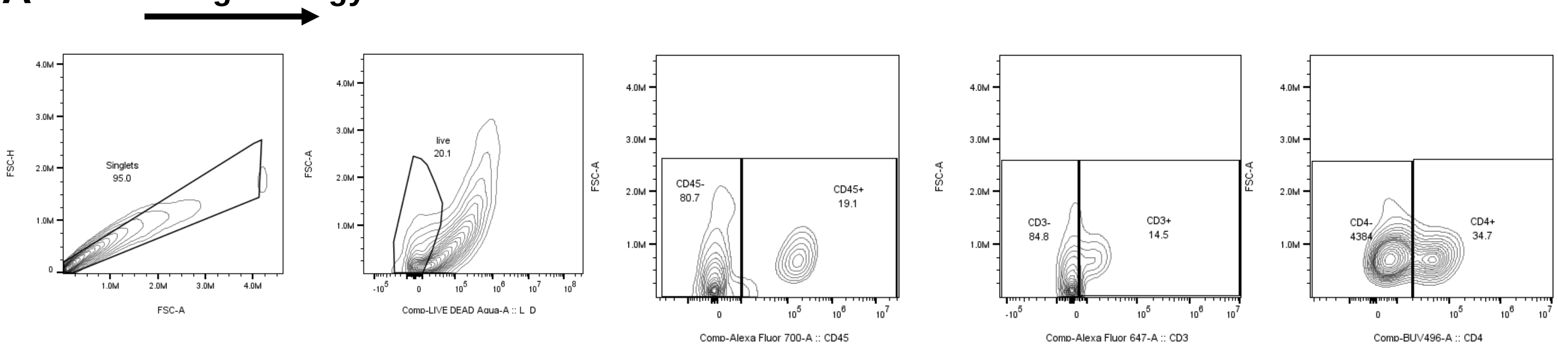

**Supplemental Figure 4: Tregs from EphB4 KD tumors downregulate Th1 and Th2 differentiation and mTOR signaling *in vivo*.**

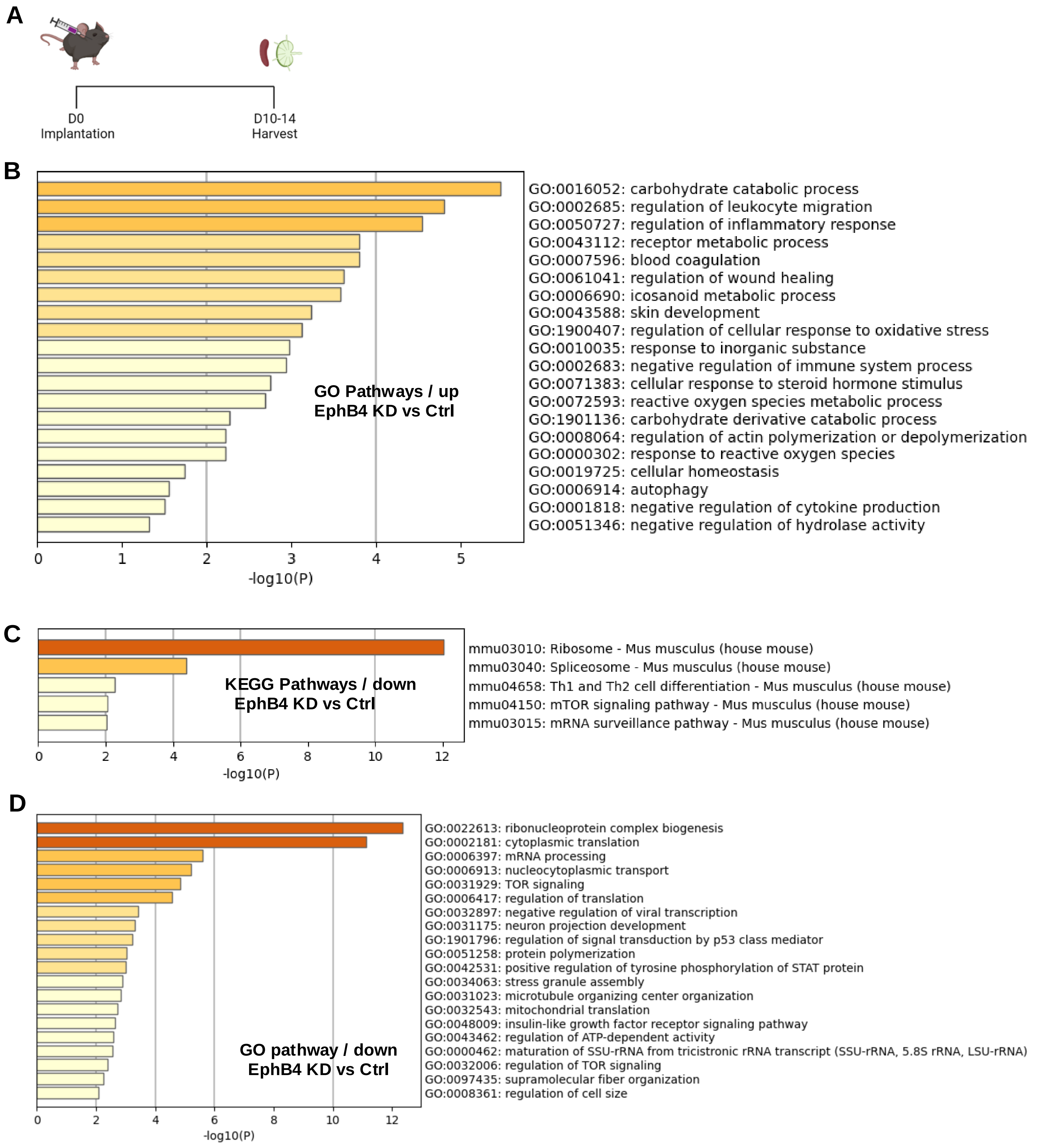

**Supplemental Figure 5: EphrinB2 KO in vascular endothelial cells coupled with radiation therapy reduces local tumor growth.**

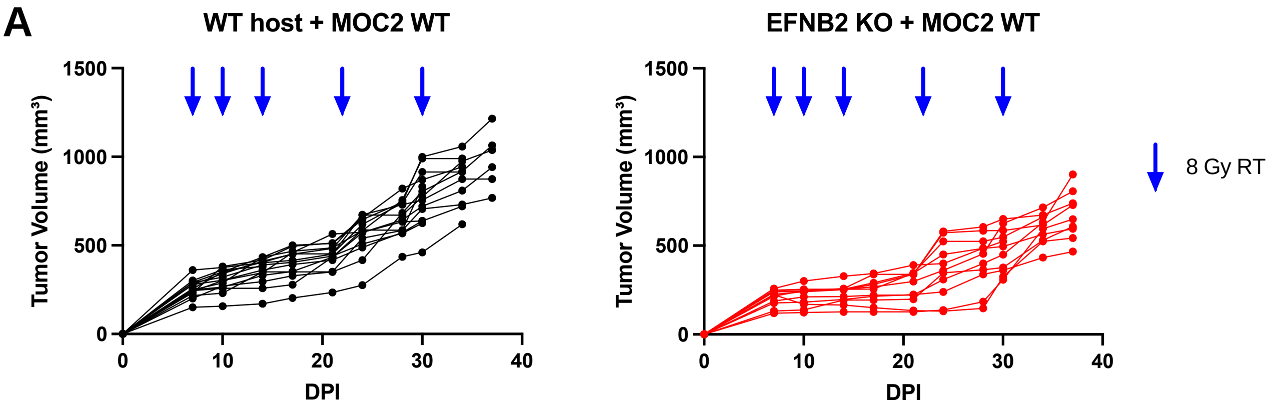

**Supplemental Figure 6: EphrinB2 expression in vascular endothelial cells increases after RT.**

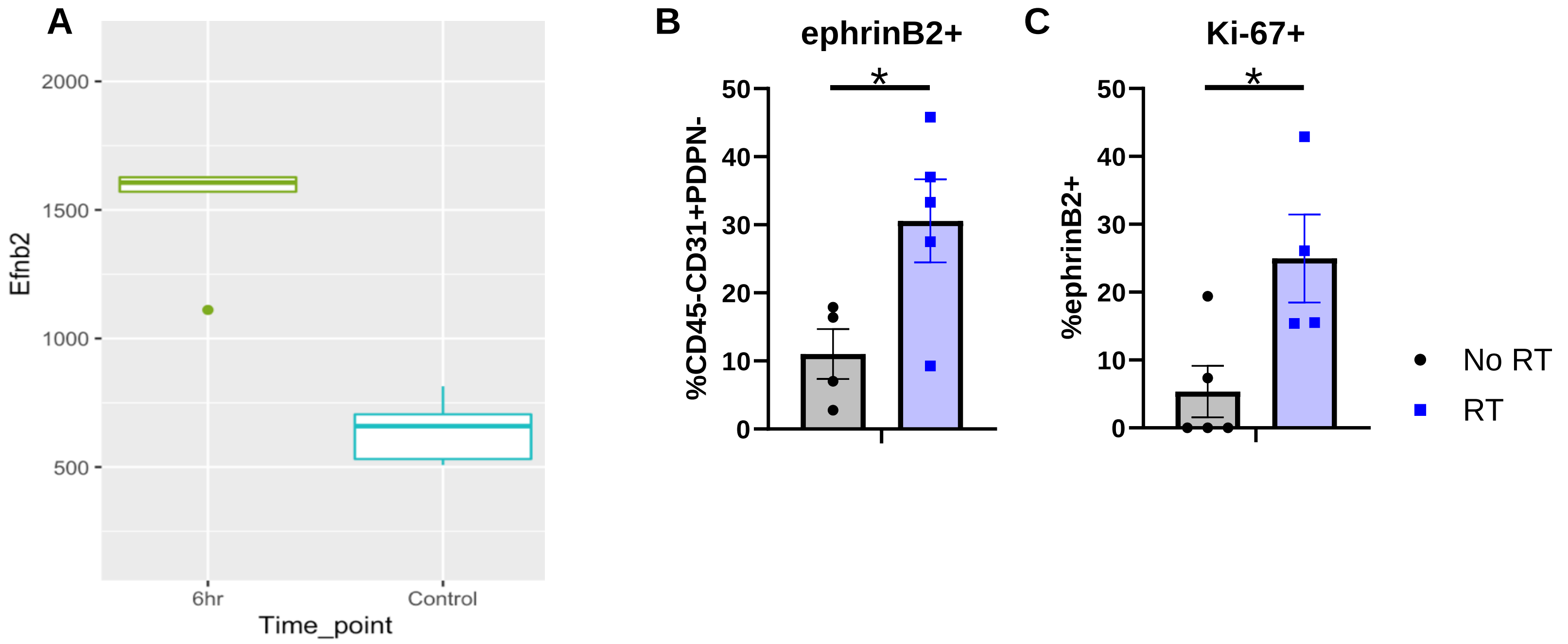

# Supplemental Figure 7: Gating strategy for immune cell populations in the TME of ephrinB2 KO mice.

## A

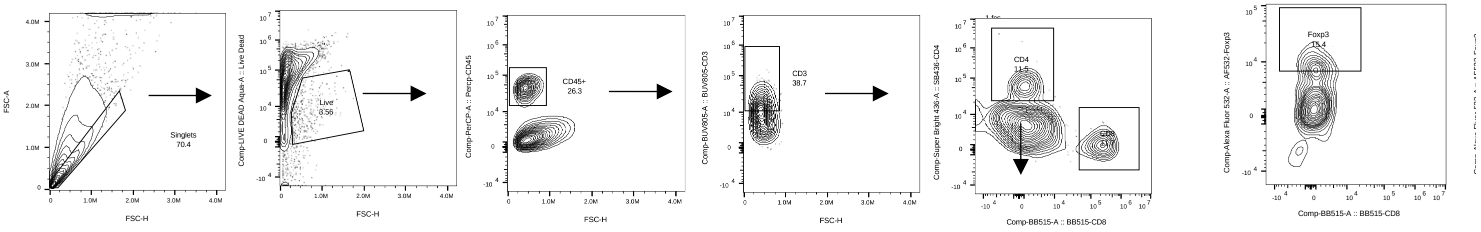

# Supplemental Figure 8: EphrinB2 KO in vascular endothelial cells affects the systemic immune response.

## A Blood gating

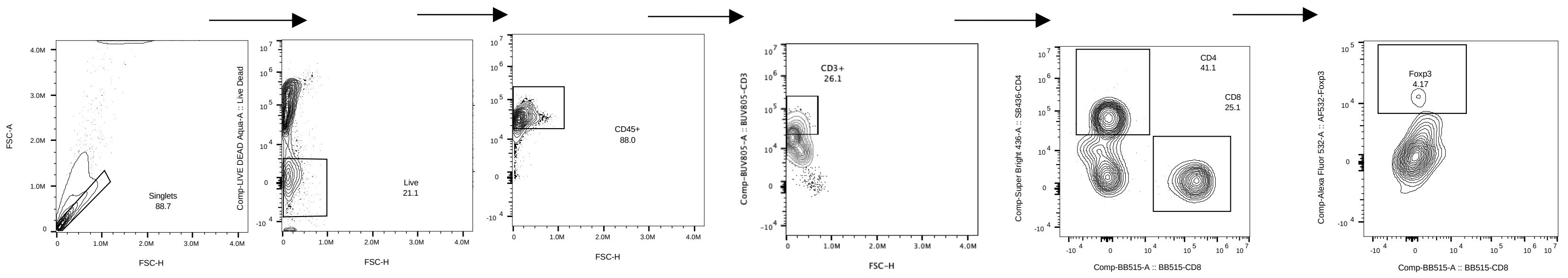


## B

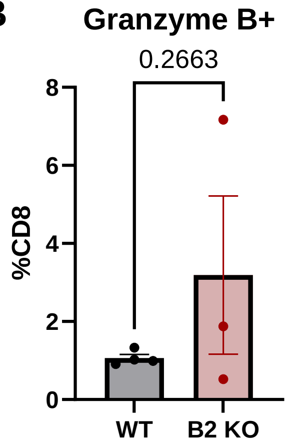


## C DLN gating

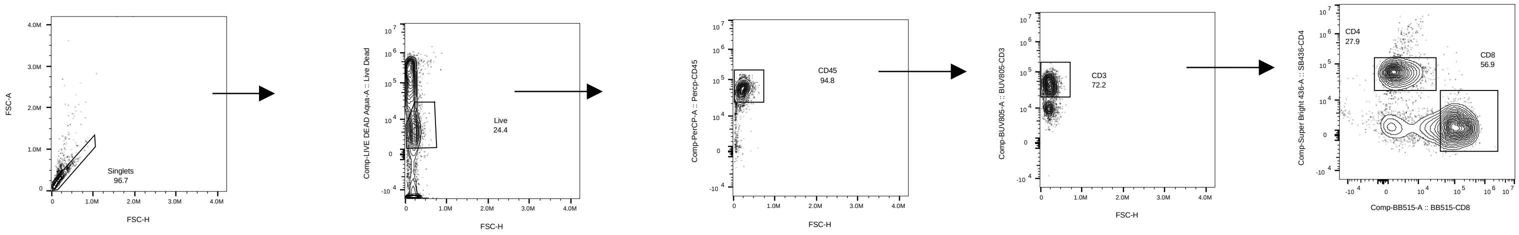


## D

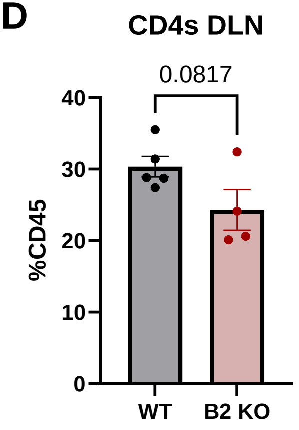


## E

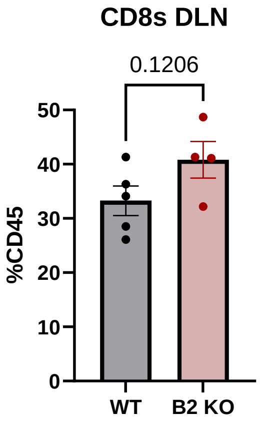


## F

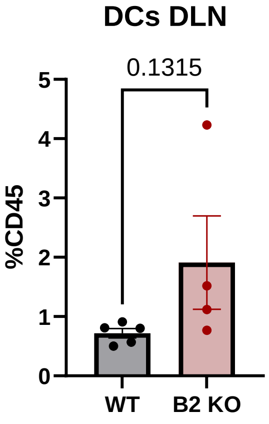


## G

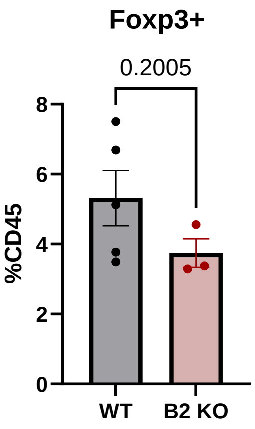

**Supplemental Figure 9: Specialized flow cytometry demonstrates increased CD4 T cell accumulation in the TME of ephrinB2 KO mice.**

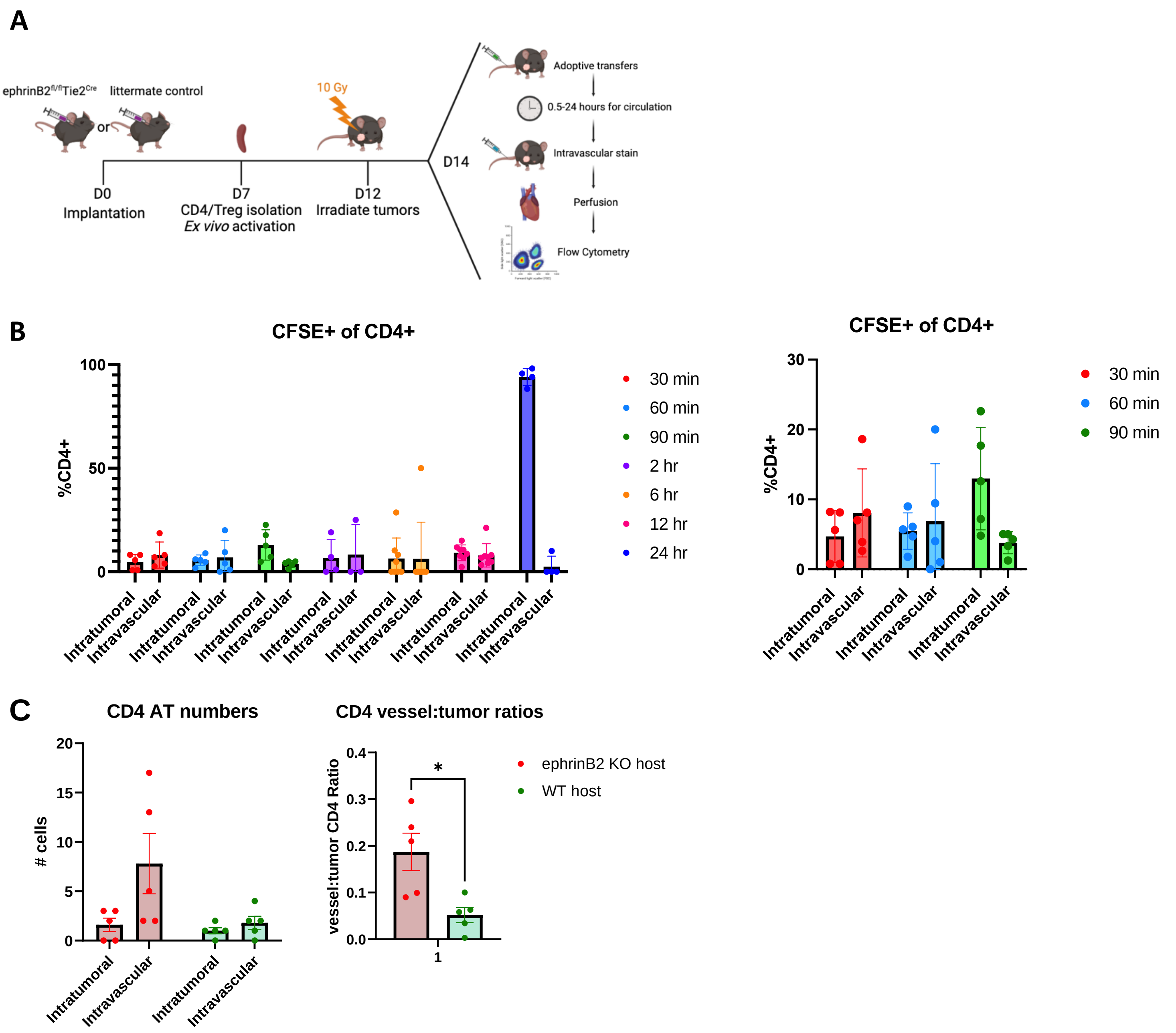

**Supplemental Figure 10: EFNB2-Fc-His and Fc-TNYL-RAW-GS reduce local tumor growth.**

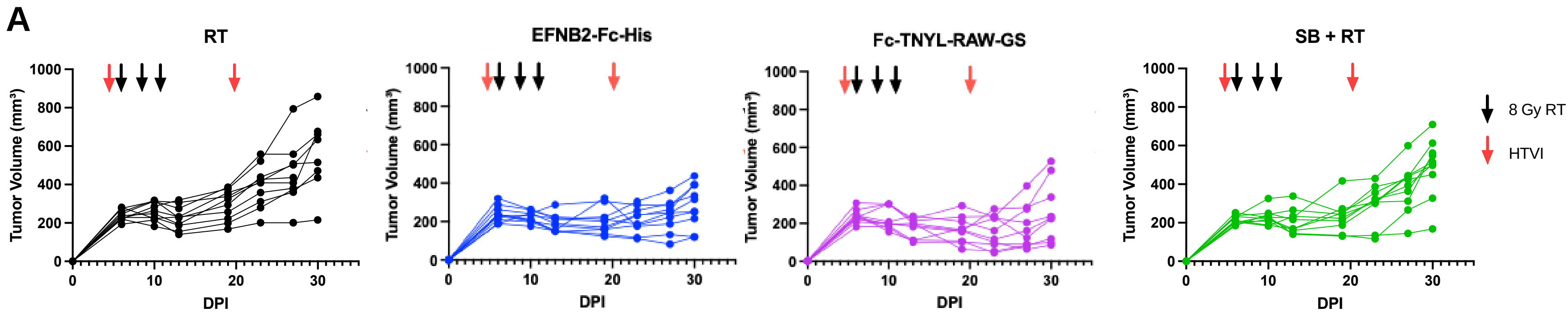

**Supplemental Figure 11: Detection of Fc fusion proteins in mouse serum and EphB4 phosphorylation following treatment with EFNB2-Fc-His and Fc-TNYL-RAW-GS.**

A

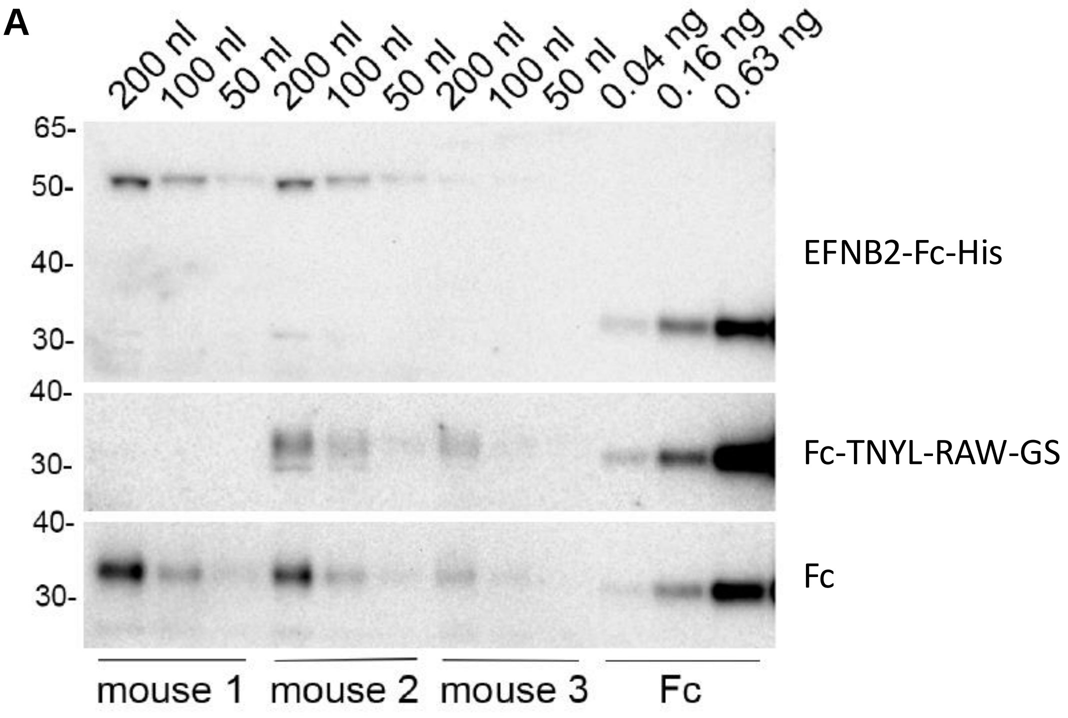


B

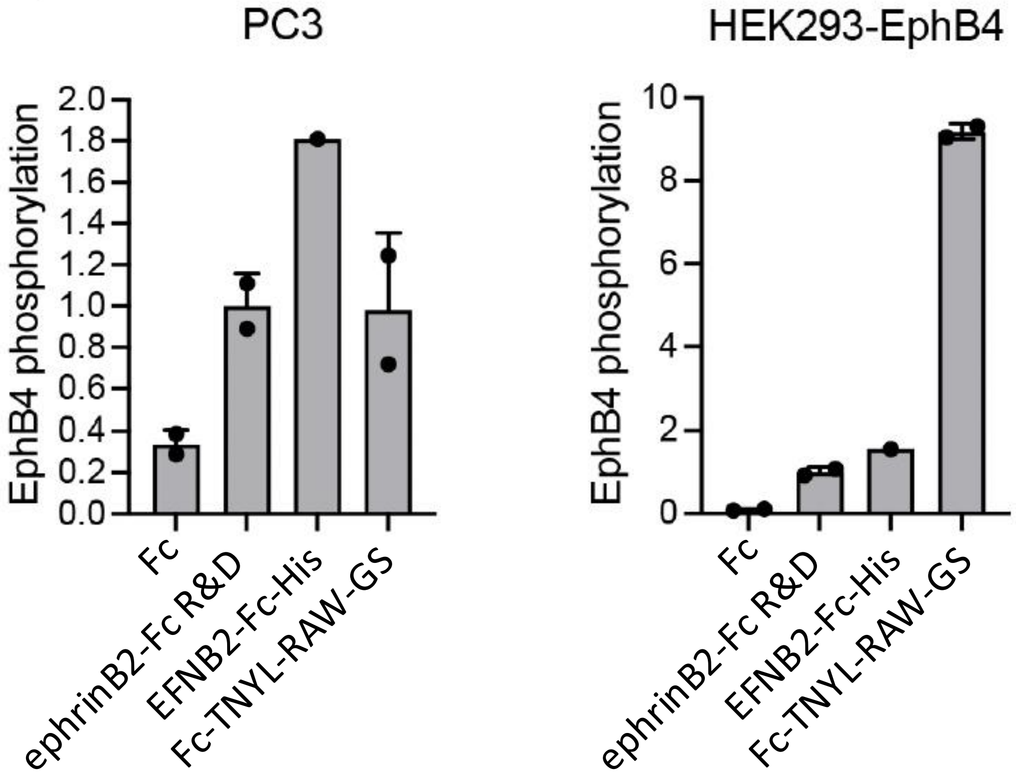

Supplement: Supplementary file 1 — Supplemental Figures [file 41388_2024_3208_MOESM1_ESM.pdf]
