# Supplementary material for: Manipulating the EphB4-ephrinB2 axis to reduce metastasis in HNSCC
Source: Oncogene. 2024 Nov 3;44(3):130–46. doi: 10.1038/s41388-024-03208-9 (PMC11725500; doi:10.1038/s41388-024-03208-9)
Supplement: Supplementary file 2 — Supplemental Figure Legends [file 41388_2024_3208_MOESM2_ESM.pdf]

Supplemental Figure Legends

Supplemental Figure 1: Loss of EphB4 in cancer cells significantly increases local tumor growth in the context of radiotherapy. (A) Average tumor volume curves comparing control shRNA versus EphB4 shRNA MOC2 tumors implanted in C57BL/6 mice ($n = 10$ per group). (B) Spaghetti plots of tumor volume for C57BL/6 mice implanted with MOC2 Ctrl (black) or EphB4 shRNA (red) tumors. (C) Dot plot showing significantly larger average tumor volume at 35 DPI in MOC2 EphB4 shRNA implanted C57BL/6 mice compared to controls. (D) Average tumor volume curves comparing Ctrl shRNA versus EphB4 shRNA LY2 cancer cells implanted in BALB/c mice ($n = 10$ per group). (E) Spaghetti plots of tumor volume for BALB/c mice implanted with LY2 Ctrl (black) or EphB4 shRNA (red) tumors. (F) Dot plot showing significantly larger average tumor volume at 20 DPI in LY2 EphB4 shRNA implanted BALB/c mice compared to controls. (G) Representative 3D lung contouring generated using ITK-Snap from CT scans collected over time for MOC2 implanted C57BL/6 mice. Lung lesions are shown in blue. (H) Hematoxylin and eosin staining of lung tissue for Ctrl and EphB4 shRNA MOC2 implanted C57BL/6 mice. Comparison of tumor volume between the control and experimental group was done using two-sided student's *t*-test. Significance was determined if the *p*-value was <0.05*, <0.01**, <0.001***, and <0.0001****. *p*-values are indicated for the figures C ****$p < 0.0001$, F *$p = 0.0002$. The error bars represent the standard error of the mean (± SEM).

Supplemental Figure 2: EphB4 KO cancer cells express increased E-cadherin, Wnt-5a, and Wnt7a as well as decreased vimentin *in vitro* and *in vivo*. Mass spectrometry proteomics data showing increased E-cadherin and decreased vimentin in MOC2 EphB4 KO cancer cells (A) ($n = 3$ per group) and tumors (B) ($n = 5$ per group) compared to controls. (C) Mass spectrometry proteomics data showing upregulation of Wnt5a and Wnt7a protein expression in MOC2 EphB4 KO tumors compared to controls ($n = 3$ per group). The experiments were performed once with their own biological replicates. Comparison of relative protein expression between the control and experimental groups was done using two-sided student's *t*-test. *p*-values are indicated for the figures A E-Cadherin ***$p = 0.0008$; vimentin

*$p = 0.0241$, C Wnt-5a **$p = 0.0042$; Wnt7a **$p = 0.0049$. The error bars represent the standard error of the mean (± SEM).

Supplemental Figure 3: Gating strategy for cancer cells and CD4+ T cell coculture experiment. (A) Gating strategy for flow conducted after coculture of LY2 Ctrl or EphB4 shRNA cancer cells with CD4+ T cells.

Supplemental Figure 4: Tregs from EphB4 KD tumors downregulate Th1 and Th2 differentiation and mTOR signaling *in vivo*. (A) Schematic experimental design for C57BL/6 mice implanted with MOC2 Ctrl or EphB4 shRNA tumors ($n = 5$ per group). Tregs were isolated from spleens and lymph nodes using a MACS Miltenyi Biotec CD4+CD25+ Regulatory T Cell Isolation Kit, and cells were subsequently flow sorted by CD25+ expression. B) Bulk proteomic pathway analyses showing GO processes that were upregulated in Tregs of MOC2 EphB4 shRNA tumors compared to controls. (C) Bulk proteomic pathway analyses showing KEGG pathways that were downregulated in Tregs of MOC2 EphB4 shRNA tumors compared to controls. (D) Bulk proteomic pathway analyses showing GO processes that were downregulated in Tregs of MOC2 EphB4 shRNA tumors compared to controls. The experiments were performed once with their own biological replicates.

Supplemental Figure 5: EphrinB2 KO in vascular endothelial cells coupled with radiation therapy reduces local tumor growth. (A) Spaghetti plots comparing tumor volume between WT hosts ($n = 14$) and ephrinB2$^{fl/fl}$Tie2^{Cre} mice ($n = 11$) implanted with MOC2 WT tumors.

Supplemental Figure 6: EphrinB2 expression in vascular endothelial cells increases after RT. (A) RNA expression of ephrinB2 in sorted VE-Cadherin+ vascular endothelial cells 6 hours after 5 Gy total body irradiation of C57BL/6 mice. (B) Quantification of ephrinB2-expressing vascular endothelial cells (CD45-CD31+PDPN-) in the TME of MOC2 tumors treated with ($n = 4$) or without ($n = 5$) radiation. (C) Quantification of Ki-67 expression in ephrinB2-expressing vascular endothelial cells in the MOC2 TME (No RT, $n = 5$; RT, $n = 4$). 10 Gy was administered 7 DPI, and tissue was harvested 17 DPI. The

experiments were performed once with their own biological replicates. Comparison of differences between the control and experimental group was done using a two-sided student's *t*-test. Significance was determined if the *p*-value was <0.05*, <0.01**, <0.001***, and <0.0001****. *p*-values are indicated for the figures B *p = 0.0373, C *p = 0.0285**.** The error bars represent the standard error of the mean (± SEM).

Supplemental Figure 7: Gating strategy for immune cell populations in the TME of ephrinB2 KO mice. (A) Flow cytometry gating strategy for tumor immune cell populations in WT hosts or ephrinB2$^{fl/fl}$Tie2^{Cre} mice implanted with 100k MOC2 WT cancer cells in the buccal mucosa.

Supplemental Figure 8: EphrinB2 KO in vascular endothelial cells affects the systemic immune response. (A) Flow cytometry gating strategy for the blood compartment. (B) Quantification of granzyme B expressing CD8+ T cells in the blood (WT, n = 4; B2 KO, n = 3). (C) Flow cytometry gating strategy for the DLN compartment. Quantification of CD4+ T cells (D), CD8+ T cells (E), and dendritic cells (F) in the DLNs (WT, n = 5; B2 KO, n = 4). (G) Quantification of Tregs in the DLNs (WT, n = 5; B2 KO, n = 3). Comparison of differences between the control and experimental group was done using a two-sided student's *t*-test. Significance was determined if the *p*-value was <0.05*, <0.01**, <0.001***, and <0.0001****. The error bars represent the standard error of the mean (± SEM).

Supplemental Figure 9: Specialized flow cytometry demonstrates increased CD4 T cell accumulation in the TME of EFNB2 KO mice. (A) Schematic of flow cytometry extravasation experiments designed to examine CD4+ T cell accumulation into the TME. EphrinB2$^{fl/fl}$Tie2^{Cre} mice or WT controls were implanted with 100k MOC2 EphB4 shRNA cells. 14 days post-implantation, mice were irradiated with 10 Gy. 2 days later, 10 million CD4 T cells were stained and adoptively transferred. At various timepoints following adoptive transfers, tail vein injections of CD4-APC were administered to label vascular immune cells. After 3 minutes, mice were euthanized, and their hearts were perfused to flush out excess stain. Tumors were subsequently harvested for flow cytometric analysis. (B) Quantification of intravascular and intratumoral CD4+ T cells at various timepoints following adoptive

transfers. Graph on the left shows a close-up view of shorter timepoints where tumors were harvested 30, 60, and 90 minutes following adoptive transfer (n = 5 per group). (C) Quantification and ratios of intravascular and intratumoral CD4 T cells 1 hour following adoptive transfer into ephrinB2$^{fl/fl}$Tie2^{Cre} (ephrinB2 KO) or WT hosts implanted with EphB4 shRNA tumors (n = 5 per group). The experiments were performed once with their own biological replicates. Comparison of differences between the control and experimental group was done using a two-sided student's t-test. Significance was determined if the p-value was <0.05*, <0.01**, <0.001***, and <0.0001****. p-values are indicated for the figures C *p = 0.0141. The error bars represent the standard error of the mean (± SEM).

Supplemental Figure 10: EFNB2-Fc-His and Fc-TNYL-RAW-GS reduce local tumor growth. (A) Spaghetti plots comparing tumor volume among C57BL/6 mice implanted with MOC2 WT cancer cells, treated with 8 Gy RT, and transfected with plasmids by hydrodynamic tail vein injections (n = 10 per group). Mice treated with RT alone (black) or SB + RT (green) served as controls. Experimental mice were treated with EFNB2-Fc-His (blue) or Fc-TNYL-RAW-GS (purple).

Supplemental Figure 11: Detection of Fc fusion proteins in mouse serum and EphB4 phosphorylation following treatment with EFNB2-Fc-His and Fc-TNYL-RAW-GS. (A) Blood was collected from tumor-bearing mice 26 days after plasmid hydrodynamic tail vein injection and the indicated nanoliters (nl) of serum were separated by SDS-PAGE and probed by immunoblotting with anti-human Fc antibodies. The indicated amounts of purified human Fc were also included for comparison and to enable approximate quantification of the Fc fusion proteins from mouse blood. Serum was obtained from 3 mice injected with each construct. (B) ELISA measuring EphB4 tyrosine phosphorylation induced by 2.5 nM purified commercial ephrinB2-Fc (ephrinB2-Fc R&D) (n = 2), EFNB2-Fc-His (n = 1), Fc-TNYL-RAW-GS peptide (n = 2), or Fc purified from the culture medium of transfected HEK293 cells (n = 2). Phosphorylation was measured for endogenous EphB4 expressed in PC3 prostate cancer cells (left) or EphB4 stably expressed in HEK293 cells (right). Fc was used as a control. The experiments were performed once with their own biological replicates.
